# The Slugs of Britain and Ireland: Undetected and Undescribed Species Increase a Well-Studied, Economically Important Fauna by More Than 20%

**DOI:** 10.1371/journal.pone.0091907

**Published:** 2014-04-16

**Authors:** Ben Rowson, Roy Anderson, James A. Turner, William O. C. Symondson

**Affiliations:** 1 National Museum of Wales, Cardiff, Wales, United Kingdom; 2 Conchological Society of Great Britain & Ireland, Belfast, Northern Ireland, United Kingdom; 3 Cardiff School of Biosciences, Cardiff University, Cardiff, Wales, United Kingdom; Australian Museum, Australia

## Abstract

The slugs of Britain and Ireland form a well-studied fauna of economic importance. They include many widespread European species that are introduced elsewhere (at least half of the 36 currently recorded British species are established in North America, for example). To test the contention that the British and Irish fauna consists of 36 species, and to verify the identity of each, a species delimitation study was conducted based on a geographically wide survey. Comparisons between mitochondrial DNA (COI, 16S), nuclear DNA (ITS-1) and morphology were investigated with reference to interspecific hybridisation. Species delimitation of the fauna produced a primary species hypothesis of 47 putative species. This was refined to a secondary species hypothesis of 44 species by integration with morphological and other data. Thirty six of these correspond to the known fauna (two species in Arion subgenus Carinarion were scarcely distinct and Arion (Mesarion) subfuscus consisted of two near-cryptic species). However, by the same criteria a further eight previously undetected species (22% of the fauna) are established in Britain and/or Ireland. Although overlooked, none are strictly morphologically cryptic, and some appear previously undescribed. Most of the additional species are probably accidentally introduced, and several are already widespread in Britain and Ireland (and thus perhaps elsewhere). At least three may be plant pests. Some evidence was found for interspecific hybridisation among the large Arion species (although not involving A. flagellus) and more unexpectedly in species pairs in Deroceras (Agriolimacidae) and Limacus (Limacidae). In the latter groups, introgression appears to have occurred in one direction only, with recently-invading lineages becoming common at the expense of long-established or native ones. The results show how even a well-studied, macroscopic fauna can be vulnerable to cryptic and undetected invasions and changes.

## Introduction

Slugs are among the invertebrates most readily encountered by people in north-west Europe, with many species occurring in gardens and in or around buildings. The British and Irish slug fauna of 36 species [1] includes important pests, post-glacial relicts, indicators of ancient woodland and even putative endemics [2]. However, most are widespread European species, many of them introduced in other parts of the world. Almost all the introduced slugs of other temperate regions also occur in Britain, including those of South Africa (11 species [3]) and New Zealand (14 species [4]). At least 18 species (half the currently recognised British fauna) are established in the USA and/or Canada [5], [6], [7]. Some of these species have a long history of study. The early depictions of British slugs in Lister (1685) [8] were a source for the descriptions by Linnaeus (1758) [9] and other early European workers. After Scharff's (1891) Irish monograph [10], the British fauna was monographed twice in the 20^th^ century [11], [12], establishing a benchmark for identification guides [13], [14], population genetic studies (e.g. [15], [16], [17]) and applied works (e.g. [18], [19]). In Britain and Ireland, slugs have been included in a pioneering mollusc distribution mapping scheme since the 1880s [2] so have been subject to careful public-participatory recording and study, resulting in updated and comprehensive checklists (most recently in 2008 [1]). As a result, the British and Irish slug fauna must rank among the world's best studied.

However, additional species in the fauna have nevertheless been recognised relatively recently either by examining “aggregates” of superficially similar species [20], [21] or direct detection [22], [23], [24]. Wide-ranging phylogeographic work has also demonstrated the presence of additional taxa in Britain [25]. In order to detect such taxa they must be distinguished from those already known to be present, which must themselves be adequately characterised. Slugs present particular problems in identification due to overlapping external morphology and the need to examine internal characters, so despite their importance and conspicuousness they are often neglected during biodiversity assessments and also by amateur malacologists. In 2011 we began producing a new comprehensive identification guide to the British and Irish slugs, aimed at non-specialists (the most recent being [14]). Such guides depend upon correctly identified reference specimens, ideally vouchered in an accessible museum collection. In the case of slugs these should preferably be photographed alive since some diagnostic features are lost or obscured on preservation. We took this opportunity to sequence mitochondrial DNA (mtDNA) from these and other specimens. This provided an independent corroborator of identifications where conspecific reference sequences already existed, or a potential future reference where they did not.

The survey also allowed us to use species delimitation techniques to test the contention that there are currently 36 slug species established in Britain and Ireland [1]. “Established” refers to reproducing populations (whether native or introduced) as opposed to “adventive” populations that do not sustain themselves by reproduction. Invertebrate “species” in a checklist such as [1] have usually been delimited, studied and recorded by non-molecular techniques (*Arion (Mesarion) fuscus* (Müller) is an exception [26]). In general, they have been considered species under a biological species concept invoking reproductive isolation. This species concept is sometimes difficult to apply in European slugs, which are typically simultaneous hermaphrodites with a varying tendency to self-fertilise [16]. In the family Arionidae in particular, a flexible species concept may have to be adopted as a consequence of mixed breeding systems and variable evolutionary rates [25], [26], [27], [28], [29]. The phylogenetic species concept, where species are delimited as genetically distinct monophyletic lineages, and evolutionary species concept, where a species is a lineage with a separate evolutionary fate, have been discussed or invoked in these studies as being more appropriate. This approach is followed here. We stress that our study is an exercise in the species delimitation of the fauna of Britain and Ireland and not a phylogenetic or phylogeographic study of wide-ranging European species. However, this does not preclude species being distinguished first in an island fauna that has, through natural establishment and introduction, sampled that of the continent (e.g. [20], [23], [24]).

Sequence data from mtDNA have proven effective in the diagnosis and delimitation of European slugs as phylogenetic species, showing at least some concordance with the morphology and behaviour of species recognised by non-molecular methods and sometimes supporting their recognition as biological species also (e.g. [30], [31], [32], [33] in addition to those cited above). However, sequence data alone do not always provide sufficient objective criteria for species delimitation [34], and mtDNA has its own limitations as an evolutionary marker [35]. It is also evident that intraspecific mtDNA divergences vary considerably across terrestrial mollusc families [36]. A number of methods have recently been developed to make species delimitation from sequence data more consistent, and more independent of delimitation based on non-molecular data to which it can then be compared. Prévot et al. [37] recently applied and compared several of these in a European terrestrial mollusc with a mixed breeding system. Each delimited slightly different numbers of species, all of which were readily recognised as monophyletic on phylogenetic trees, leading them to question how a particular delimitation method might justifiably be favoured *a priori*. One method tested, Automatic Barcode Gap Discovery (ABGD) [38] has several advantages. It requires no prior knowledge of the likely intraspecific distances and is robust to their variation within a dataset. Being distance-based it does not use monophyly (allowing it to be investigated independently from distinctness) and is computationally very efficient [38]. In gastropod studies [37], [39] it delimited as few or fewer species than the coalescent-based General Mixed Yule Coalescent (GMYC) delimitation method [40], most ABGD species being a subset of those delimited by GMYC. This contrast was more marked where some species were represented by many fewer sequences than others [39]. This is a sampling problem considered an inescapable consequence of biological rarity in delimitation studies [41]. As our aims were to test a hypothesis of 36 species and to attribute specimens to these whenever justified, we used the distance-based and conservative ABGD method in our study.

To best aid future identification by confirming whether external morphological variation was intraspecific, our sampling paid more attention to the more conspicuously variable slug species, especially the “larger Arionidae” (*Arion* subgenus *Arion*). This group includes *A. (A.) ater* (Linnaeus), *A. (A.) rufus* (Linnaeus), and the notorious pest *A. (A.) vulgaris* Moquin-Tandon (called *A. lusitanicus* auct. non Mabille in some publications). These are morphologically similar species that either hybridise (e.g. [42], [17], [43], [44], [45]) or overlap morphologically to give the impression of having done so [46]. It has been suggested, at least in the British media, that they may also hybridise with *Arion (A.) flagellus* Collinge, a common species in Ireland and Britain but not elsewhere outside Iberia. The potential for hybridisation further complicates the application of species concepts in the larger Arionidae, so we sought evidence of hybridisation between these species as well as aiming to clarify their genetic identity. In the event, the data suggested that hybridisation occurs in other families.

## Materials and Methods

### Ethics statement

All necessary permits were obtained for the described study, which complied with all relevant regulations. The only protected species in this study is *Geomalacus maculosus* Allman, which occurs in the Republic of Ireland but not the UK. For this species, the necessary Licence under the Wildlife Act 1976-2010 and Derogation Licence under the European Communities (Natural Habitats) Regulations 1997 were obtained from the Department of Arts, Heritage and the Gaeltacht.

### Samples

Slugs were collected by hand, often at night and/or in wet weather, from over 200 sites across Britain (England, Scotland and Wales) and Ireland (Northern Ireland and the Republic of Ireland) between 2010 and 2012. Attempts were made to maximise the range of latitude, longitude, altitude, habitat and soil types covered. As native species were the initial focus, most collecting was done in less disturbed areas including National Nature Reserves and National Parks but we also included agricultural, urban and brownfield sites. Some known sites for the rarer species were visited repeatedly. Additional specimens were collected by members of the Conchological Society of Great Britain and Ireland and members of the public, or taken from the collections of the National Museum of Wales, Cardiff, UK, where all specimens are now deposited. The single exception was a *Testacella* specimen from the Natural History Museum, London, UK (Table S1). Specimens for sequencing were selected to cover the widest possible range of morphology, geography and habitat. This meant one or at most a few individuals from each population were selected, increasing the number of populations sampled. Adult specimens were dissected where necessary and identified with reference to the taxonomic literature and museum collections. Inevitably, a small number of juvenile slugs could only be identified tentatively by morphology and were identified *a posteriori* from the DNA results.

### Sequencing

In Arionidae, the modified universal primers 16SAR and 16SBR ([7], modified from [47]) were used to amplify an approx. 440 bp region of the 16S large subunit mitochondrial ribosomal DNA. This region has been established as the mitochondrial marker of choice in arionid studies (e.g. [27], [28], [30], [48]). For other families, the universal primers LCO1490 and HCO2198 [49] were used to amplify an approx. 650 bp region of the COI gene.

Approximately 2 mm^3^ of tail or other tissue from each specimen was incubated in 1 ml 0.1× Tris EDTA (“low TE”) at 20°C for 30 min to replace preservatives. DNA was extracted with the Qiagen DNEasy kit with a single elution with 200 μl Buffer AE. Two μl of this extract was used as template in PCRs using GE Healthcare illustra PuReTaq PCR beads with 0.25 μl of each primer (10 μM) and ultra-pure water to a volume of 25 μl. Cycling conditions (Eppendorf Mastercycler) for COI and 16S were: 94°C for 2 min 30 s, (94°C for 30 s, 47°C for 45 s, 72°C for 1 min 15 s×40 cycles), 72°C for 10 min. Conditions for ITS-1 were: 94°C for 3 min, (95°C for 1 min, 55°C for 1 min, 72°C for 2 min×35 cycles), 72°C for 5 min. After visualisation and quantification on agarose gels, products were cleaned with GE Healthcare illustra ExoStar 1-Step and sequenced by Eurofins|MWG Operon (www.eurofinsgenomics.eu). Sequences were edited and compiled in BioEdit 7.1.3 [50] and submitted to GenBank (accession numbers KF894075-KF894388, details in Table S1).

### Assembling datasets

The data were divided into subsets for analysis because: i) two different mitochondrial fragments were used; ii) several arionid subgenera or species groups have been the target of previous genetic work; iii) particular sampling attention had been paid to the larger Arionidae; and iv) several of the relevant families are not closely related to one another [51]. For 16S analysis the subsets were 1) *Arion* subgenus *Arion*, including *A. flagellus*; 2) *Arion* subgenus *Mesarion*, in which great intraspecific variability has been found [25], [26] and 3); the remaining, smaller Arionidae. Preliminary analysis indicated that *A. flagellus* could have been included in either dataset 1 or 2; the former was chosen given the suggestion that this species may hybridise with others in this dataset. Analyses were repeated on a dataset including all Arionidae. For COI data the subsets included: 4) *Limax*; and 5) Other Limacidae. Analyses were repeated on a dataset including all Limacidae. The remaining subsets were: 6) Agriolimacidae; 7) Milacidae; 8) Testacellidae; 9); Boettgerillidae; and 10) Trigonochlamydidae. All relevant sequences available from members of each family in GenBank were downloaded and incorporated into each subset (7 Oct 2013; details in Table S2) with the exception of native North American and Asian arionid genera and the eastern European limacid genus *Bielzia*. Some short *Limax* sequences showing limited overlap with our dataset, and a possibly misidentified *Deroceras* sequence (FJ917286), were excluded from further analysis. Sequences in each subset were aligned by CLUSTALW in MEGA 5.1 [52] using default parameters and checked by eye. Identical sequences were collapsed to haplotypes. Sequences were realigned for the whole family datasets for Arionidae and Limacidae.

### Analysis

Species were delimited, refined and identified in a three-stage process. Firstly, putative species were delimited statistically using the 16S and COI data to produce a primary species hypothesis for each dataset (PSH). This required no *a priori* information on the number of species expected, or how genetically different such species might be. It used a consistent approach across families and simultaneously dealt with existing GenBank data from Europe and beyond. Next, those putative species that included individuals from Britain and/or Ireland were assessed with other data to refine each PSH to a secondary species hypothesis (SSH). Finally, the species accepted in the SSH were identified by comparing them to the currently known species of the fauna [1].

To examine whether hybridisation has occurred between species-level lineages in *Arion* subgenus *Arion*, a rapidly evolving nuclear marker was also sequenced from these species. The primers ITS1 and 5.8C [25] were used to amplify an approximately 570 bp region of the internal transcribed spacer (ITS-1). These authors considered topological conflict (non-monophyly) between ITS-1 and 16S data an indicator of hybridisation and introgression between species-level lineages in subgenus *Mesarion* (see also [28], [29]).

### Primary species hypotheses

PSHs were generated with the distance-based method ABGD [38]. Briefly, this divides a set of aligned sequences into groups, statistically inferring the groups most likely to correspond to species. It does so recursively, using a range of potential maximum pairwise intraspecific distances inferred from the data. It produces an array of PSHs with different numbers of groups delimited using different distance values. For example, very small or large values inevitably delimit unrealistically many or few species [38], [39]. To avoid having to discuss all PSHs produced, some criterion is required to select which of them to investigate further. We chose the PSH corresponding to the smallest number of groups that was stable over three or more successive distance values. When the initial and recursive partitions indicated different numbers of groups at the same distance value, we chose the smaller of the two. This criterion had the advantages of being i) independent of the expected number of species; ii) consistent across different datasets; and iii) conservative in terms of the numbers of putative species recognised.

ABGD analyses were run at http://wwwabi.snv.jussieu.fr/public/abgd/abgdweb.html on aligned haplotype sequences using K2P distances. To ensure a wide range of distance values were investigated, Pmin was kept at 0.001 but the maximum potential Pmax was raised from 0.1 to 0.2. To allow comparison between a greater number of alternative distance values, the number of steps was raised from 10 to 20. Other settings were as default (relative gap width, X = 1.5; number of bins  = 20).

### Secondary species hypotheses

The SSH for each dataset consisted of an equal or smaller number of species, some putative species being combined in order to remain conservative. In the sole case where ABGD delimited a putative species in one analysis (Arionidae) but split it in the subset analysis (*Arion (Arion)*), both alternatives were considered (see Results and Discussion).

As a result of the ABGD analysis, putative species were by definition genetically distinct from their close relatives. To be considered a species under the SSH, each putative species had also to show evidence of reciprocal monophyly with respect to a sister group, so being at least a phylogenetic species. This was investigated and visualised using neighbour-joining trees based on the K2P distance, with support for monophyly quantified with 1000 bootstrap pseudoreplicates in MEGA. Gaps were treated by pairwise deletion. As a comparison using a character-based method involving a model of sequence evolution, Bayesian Inference (BI) was also used, with support for monophyly quantified with posterior probabilities. FindModel (www.hiv.lanl.gov/content/sequence/findmodel/findmodel.html) was used to select the most appropriate evolutionary model (GTR + I + Γ in each case) according to log likelihood and the Akaike information criterion. BI was implemented in MrBayes v3.1.2 [53] with two parallel runs of 5,000,000 generations, sampling trees every 1000 generations, with the first 25% of the trees discarded as burn-in and other settings as default. Convergence on a stable log likelihood before the burn-in period was evident in all analyses. These methods were also used to analyse the ITS-1 data from the *Arion (Arion)* species.

Morphological distinctness was also considered as a criterion for the recognition of species in the SSH. The external and internal (adult genital) morphology of each putative species was investigated using standard techniques. Putative species that were closely related (i.e. showed evidence of joint monophyly) but did not differ in morphology were generally not recognised as separate species in the SSH. However, it became clear that in two cases (interpreted as evidence of introgression, see below) there was conflict between putative species assignment and morphology. Thus the criterion of morphological distinctness, though useful, could not be consistently applied. To do so would also preclude the recognition of genuinely cryptic species ([54]) as SSH species, should either occur.

Refinement of PSHs to SSHs by this method is an example of integration of molecular with other data, here specifically of “integration by congruence” in the sense of Padial et al. [55]. This approach requires congruence of (for example) mtDNA and morphology for species recognition. According to Padial et al. [55], integration by congruence is less likely to overestimate species numbers and better promotes future taxonomic stability than the alternative, integration by cumulation, which requires no initial congruence between datasets.

### Comparison of the SSH to the known fauna

The species in the SSH were then compared to the species making up the known fauna of Britain and Ireland [1]. For most SSH species this was straightforward from morphological identifications already made, and/or the inclusion of GenBank sequences in each PSH. Others required recourse to museum collections and the wider taxonomic literature.

## Results and Discussion

450 sequences were obtained from 388 individuals (Table S1) and compared with 659 sequences from GenBank (Table S2), representing 695 haplotypes in all. For all three gene regions, some haplotypes were found at more than one site. The three most extreme examples were 16S haplotypes attributed to *A. (A.) flagellus*, *A. (A.) ater*, and *A. (A.) vulgaris* (22, 11 and 9 individuals respectively) that were found throughout Britain and Ireland.

### Primary species hypotheses

The PSHs generated by ABGD analyses and selected according to our criteria are summarised in Table 1. Histograms and graphs illustrating the full range of PSHs generated are given in Figs. S1–S3. Variation was too limited in Boettgerillidae and Trigonochlamydidae to conduct the analysis (mean intraspecific K2P distance 0.001 and 0.000 respectively) so each was considered to comprise a single species.

**Table 1:** Datasets and species delimitation by ABGD.

Dataset	Region	Sequences	Haplotypes	Max. prior intraspecific K2P distance over which stable	Number of putative species (total)	Number of putative species (Britain/Ireland only)
*Arion (Arion)*	16S	134	60	0.050	9	6
*Arion (Mesarion)*	16S	190	160	0.115	15	5
Other Arionidae	16S	185	96	0.038	11	9
All Arionidae	16S	509	316	0.038	34	20
*Limax*	COI	203	179	0.012	27	3
Other Limacidae	COI	72	70	0.066	6	6
All Limacidae	COI	275	249	0.028	27	9
Agriolimacidae	COI	102	80	0.022	9	7
Milacidae	COI	30	28	0.007	5	5
Testacellidae	COI	22	17	0.200	4	4
Trigonochlamydidae	COI	6	2	n/a	n/a	n/a
Boettgerillidae	COI	4	3	n/a	n/a	n/a

The maximum prior intraspecific K2P distance over which the number of putative species was stable varied considerably between taxa (0.038–0.115 in Arionidae, 0.012–0.200 in other families) (Table 1). Above this value, the number of putative species delimited fell rapidly (Figs. S1–S3). The exception was Milacidae where it rose from 5 to 8 and was again stable up to K2P 0.038, although our conservative criteria selected the former value. Compared to the whole family analysis, subset analyses generated more putative species in total in Arionidae (34 versus 35), and fewer putative species in total in Limacidae (27 versus 33). However, these differences were due to the differential splitting of continental putative species (e.g. several provisionally recognised *Limax* species). In both families, the total number of putative species occurring in Britain and Ireland was identical in the whole family and subset analyses (Table 1). The overall PSH, comprising the selected PSH from each of the subset analyses, delimited 45 putative species for Britain and/or Ireland, plus one species each from Boettgerillidae and Trigonochlamydidae.

### Secondary species hypotheses

**Figure 1:**
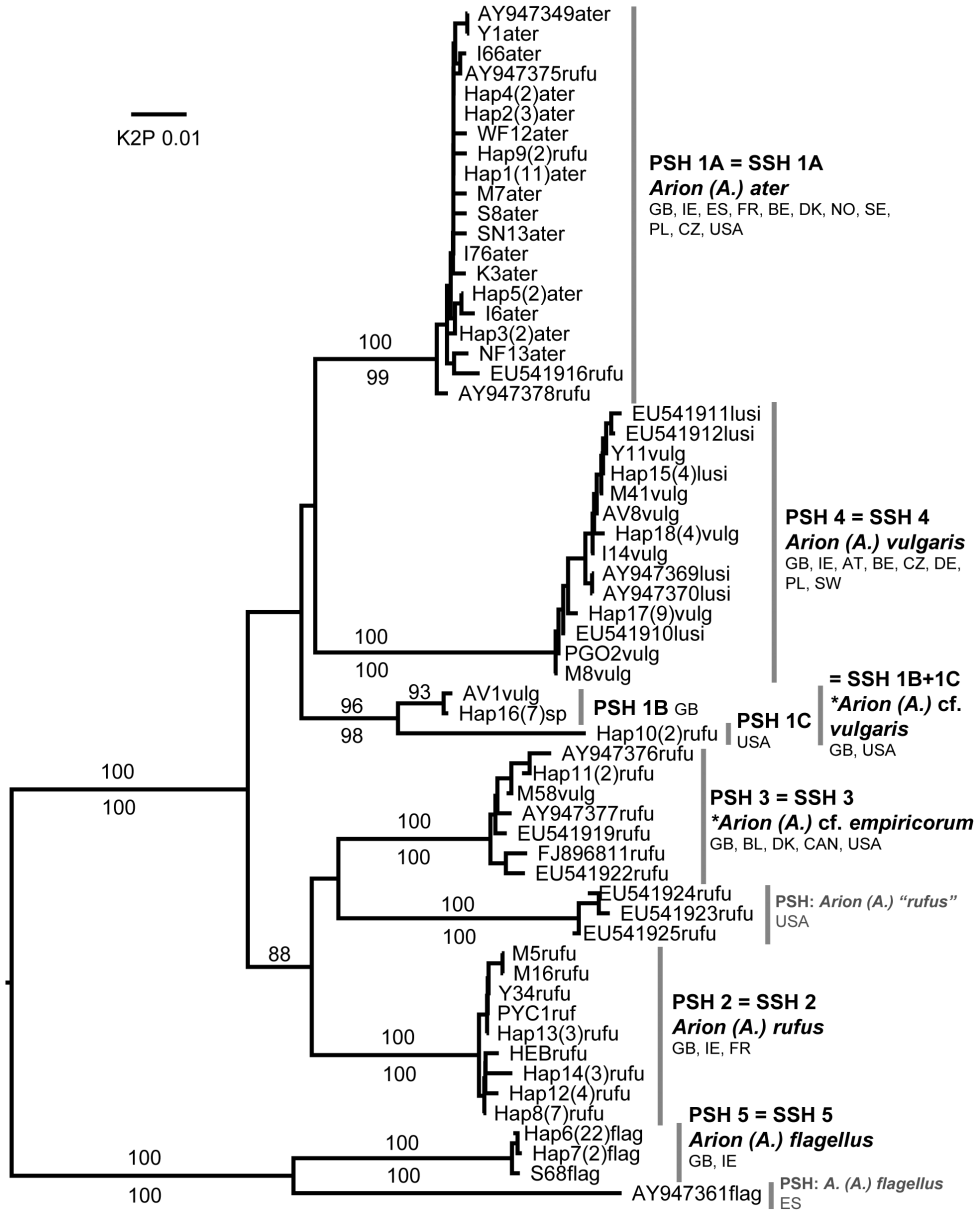
Larger Arionidae (*Arion* subgenus *Arion*). Midpoint-rooted NJ tree based on 16S data; values above branches are % bootstrap support (≥75), those below are Bayesian posterior probabilities, expressed as % (≥80). Grey bars indicate clades. Species new to the fauna of Britain and/or Ireland are indicated by *.

**Figure 2:**
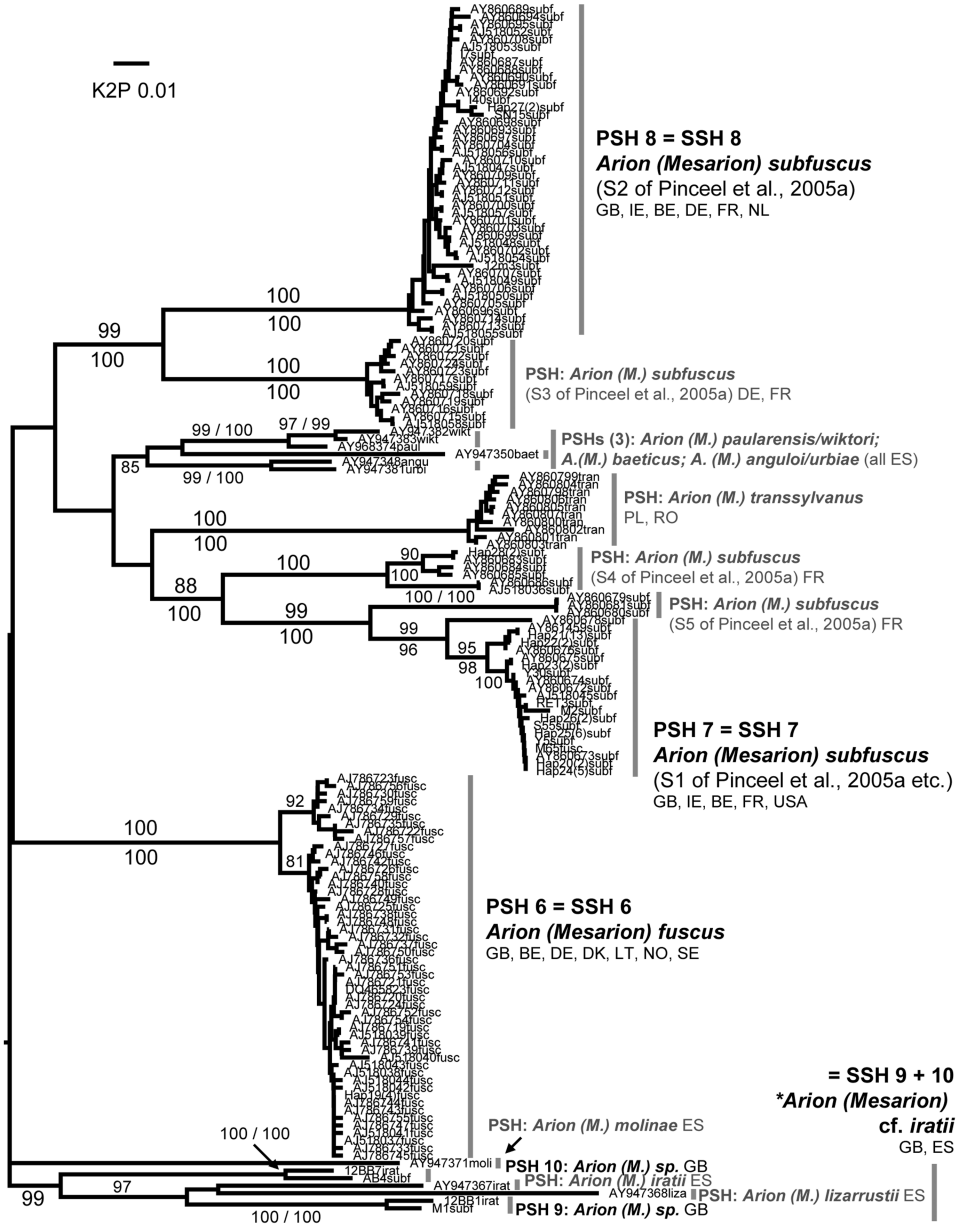
Arionidae: *Arion* subgenus *Mesarion*. Midpoint-rooted NJ tree based on 16S data; values above branches are % bootstrap support (NJ), those below are Bayesian posterior probabilities, expressed as %. Grey bars indicate clades. Species new to the fauna of Britain and/or Ireland are indicated by *.

**Figure 3:**
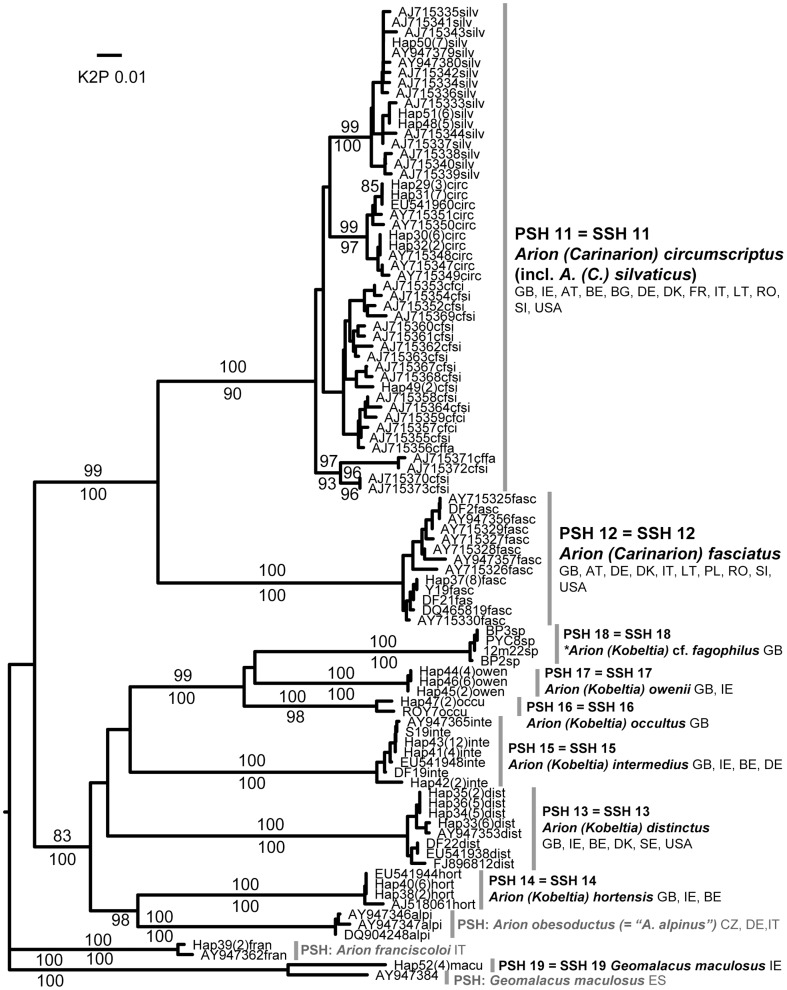
Other Arionidae. Midpoint-rooted NJ tree based on 16S data; values above branches are % bootstrap support (NJ), those below are Bayesian posterior probabilities, expressed as %. Species new to the fauna of Britain and/or Ireland are indicated by *.

**Figure 4:**
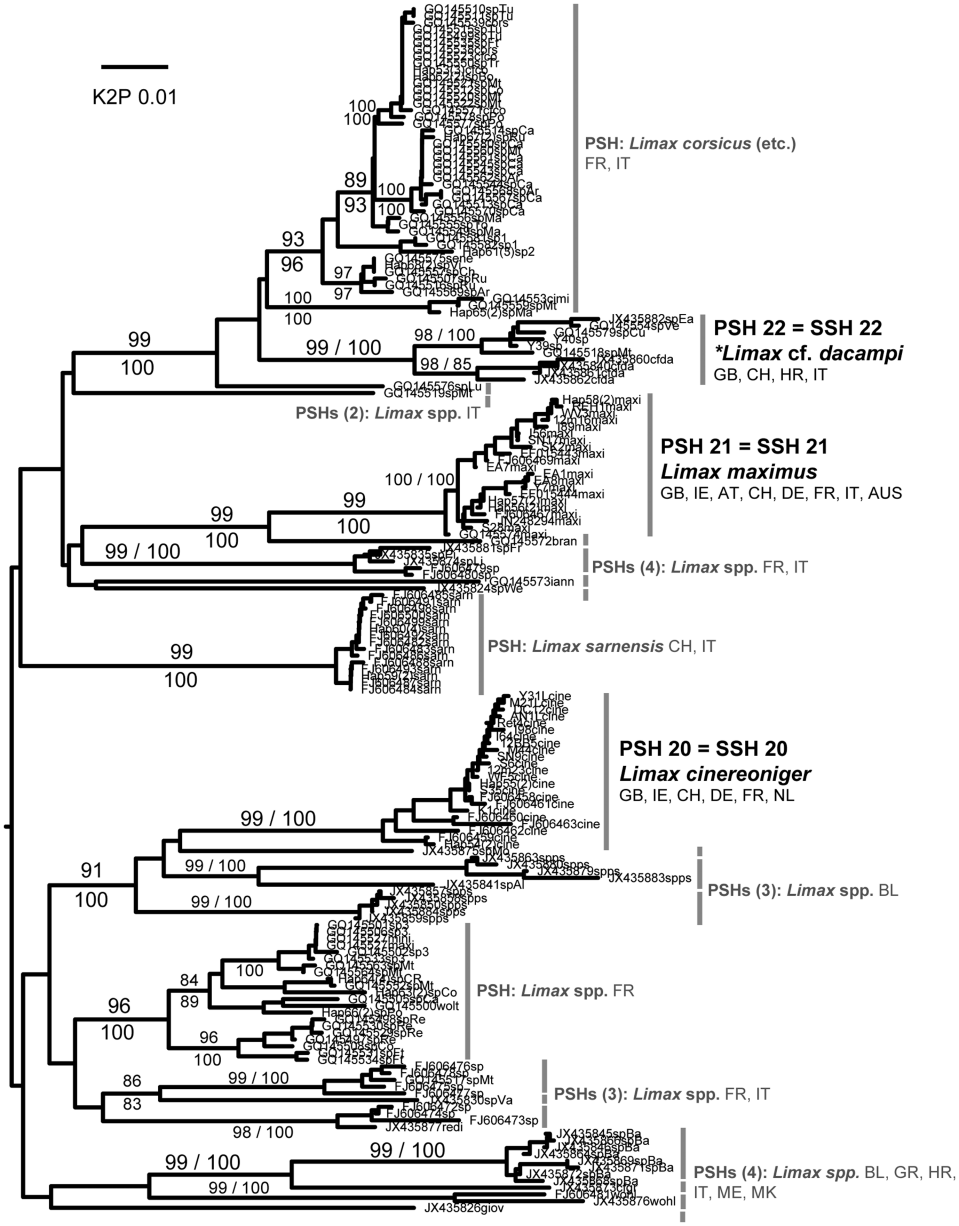
Limacidae (genus *Limax*). Midpoint-rooted NJ tree based on COI data; values above branches are % bootstrap support (NJ), those below are Bayesian posterior probabilities, expressed as %. Species new to the fauna of Britain and/or Ireland are indicated by *.

**Figure 5:**
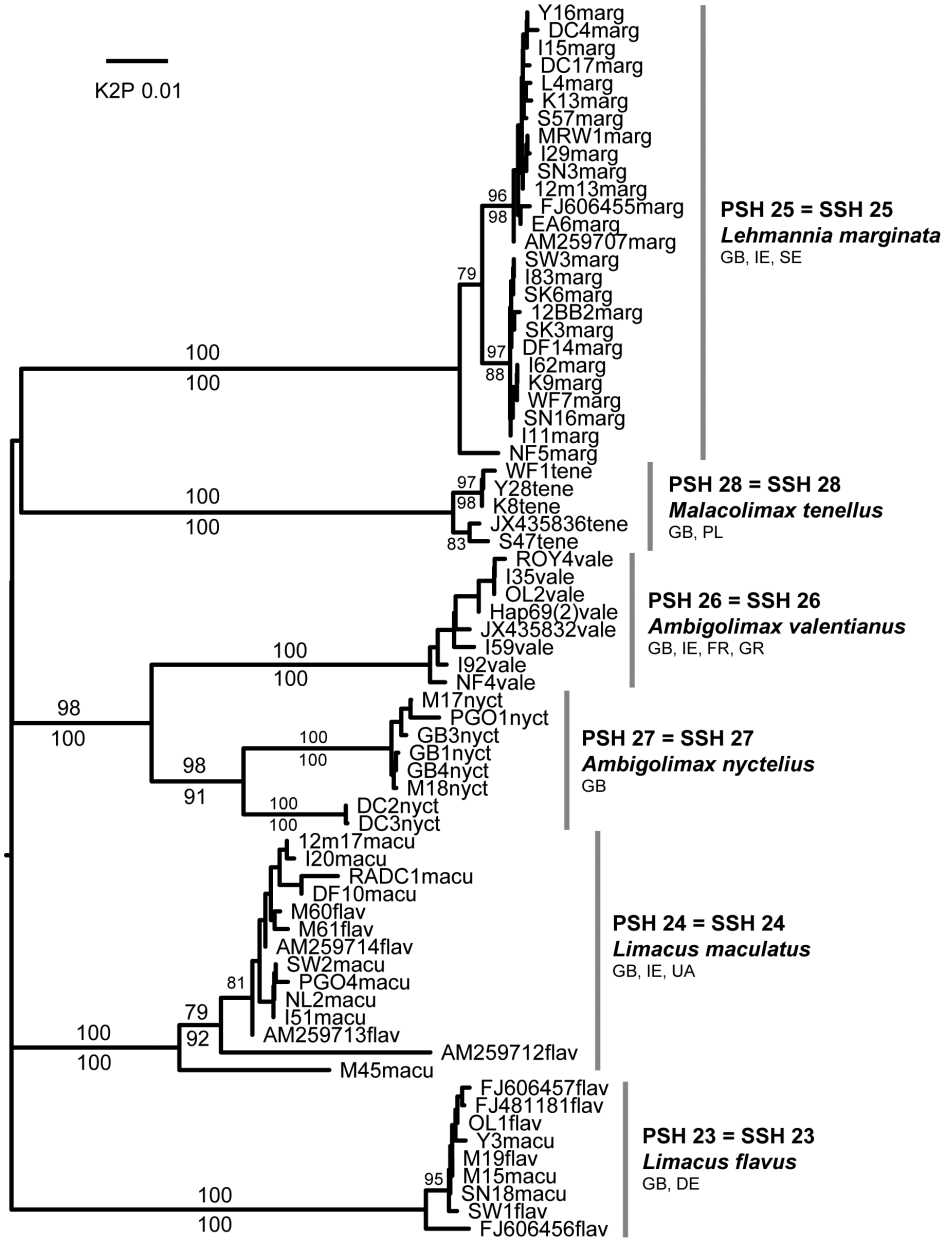
Other Limacidae. Midpoint-rooted NJ tree based on COI data; values above branches are % bootstrap support (NJ), those below are Bayesian posterior probabilities, expressed as %. Species new to the fauna of Britain and/or Ireland are indicated by *.

**Figure 6:**
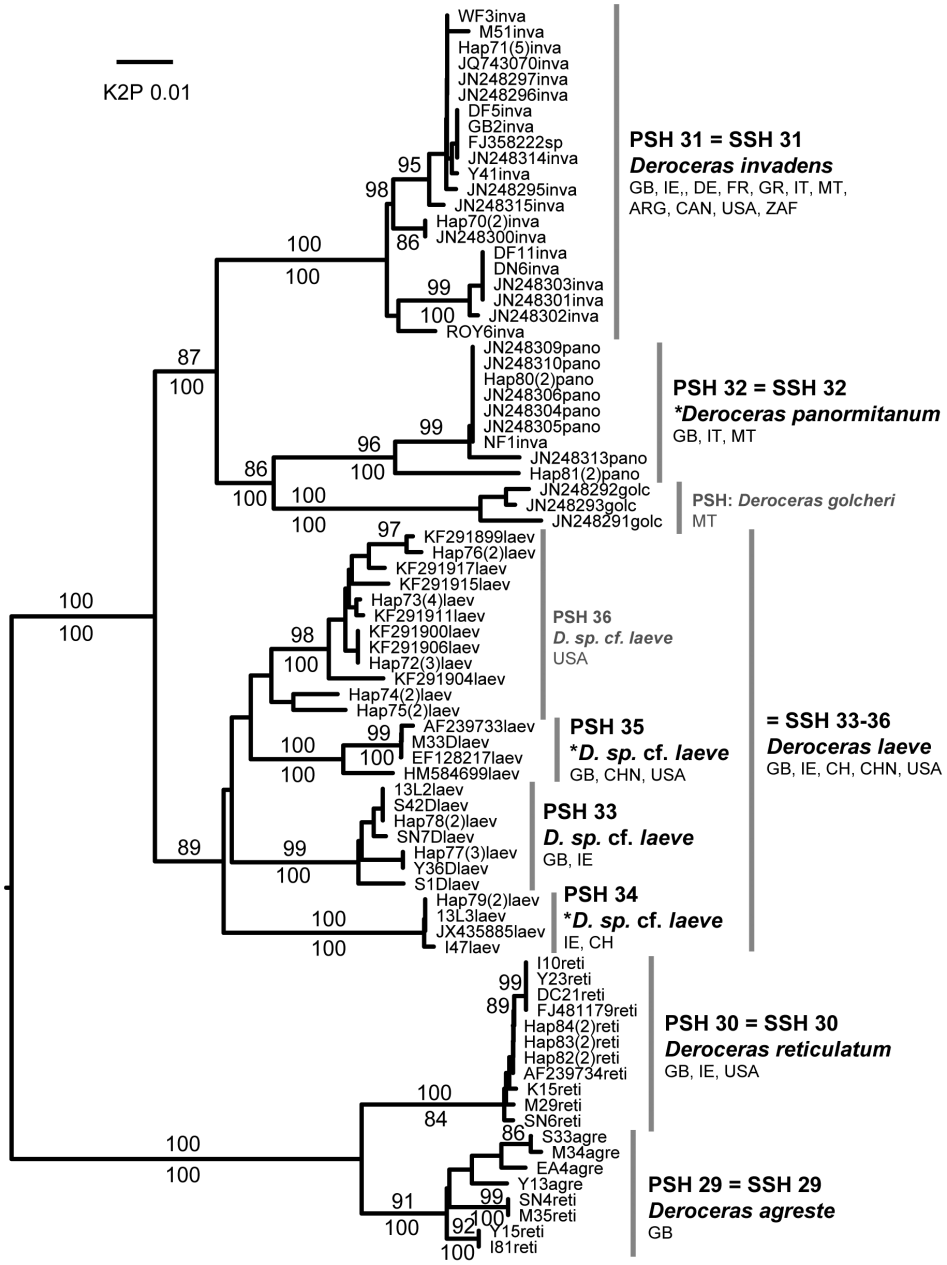
Agriolimacidae. Midpoint-rooted NJ tree based on COI data; values above branches are % bootstrap support (NJ), those below are Bayesian posterior probabilities, expressed as %. Species new to the fauna of Britain and/or Ireland are indicated by *.

**Figure 7:**
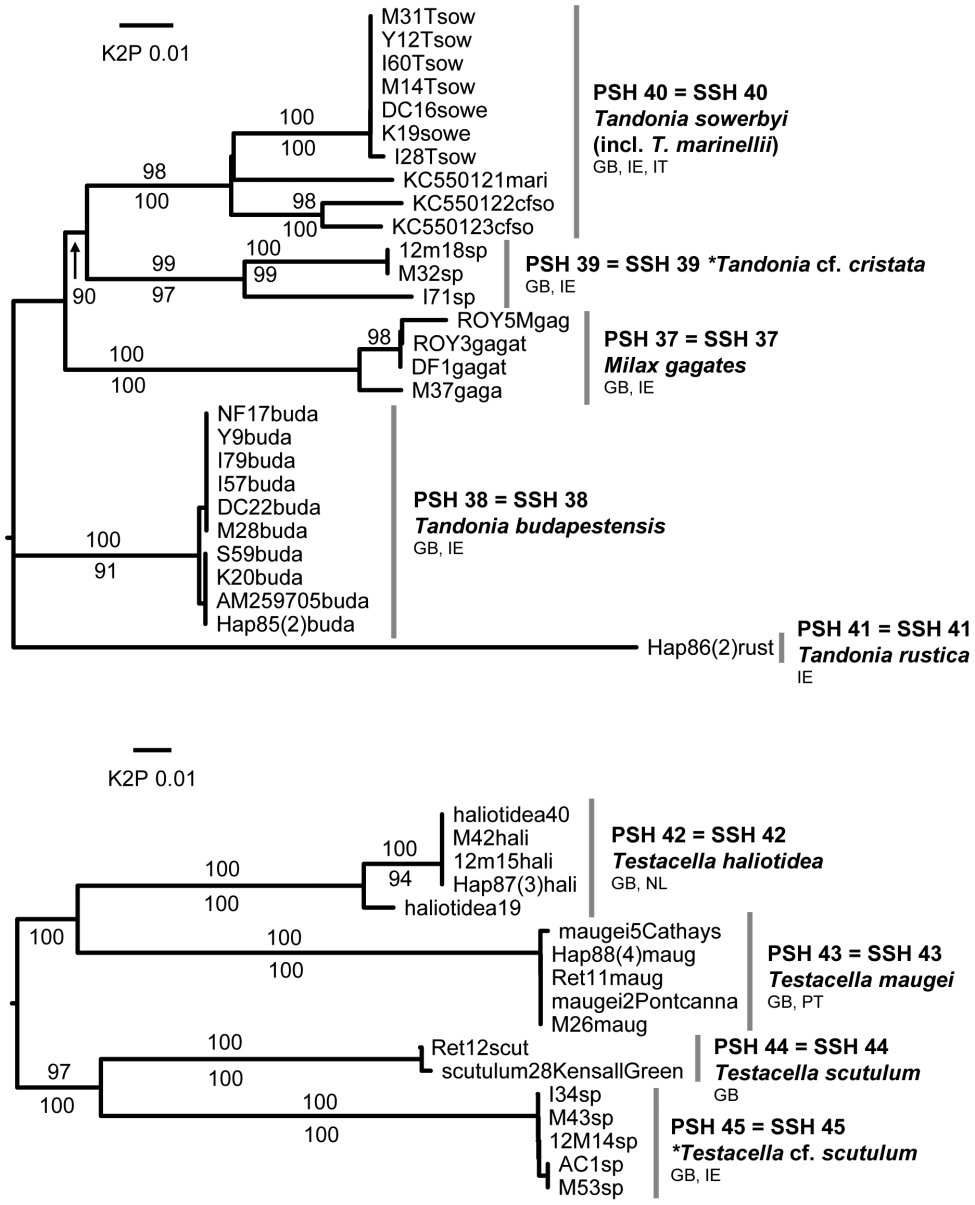
Milacidae & Testacellidae. Midpoint-rooted NJ trees based on COI data; values above branches are % bootstrap support (NJ), those below are Bayesian posterior probabilities, expressed as %. Species new to the fauna of Britain and/or Ireland are indicated by *.

**Figure 8:**
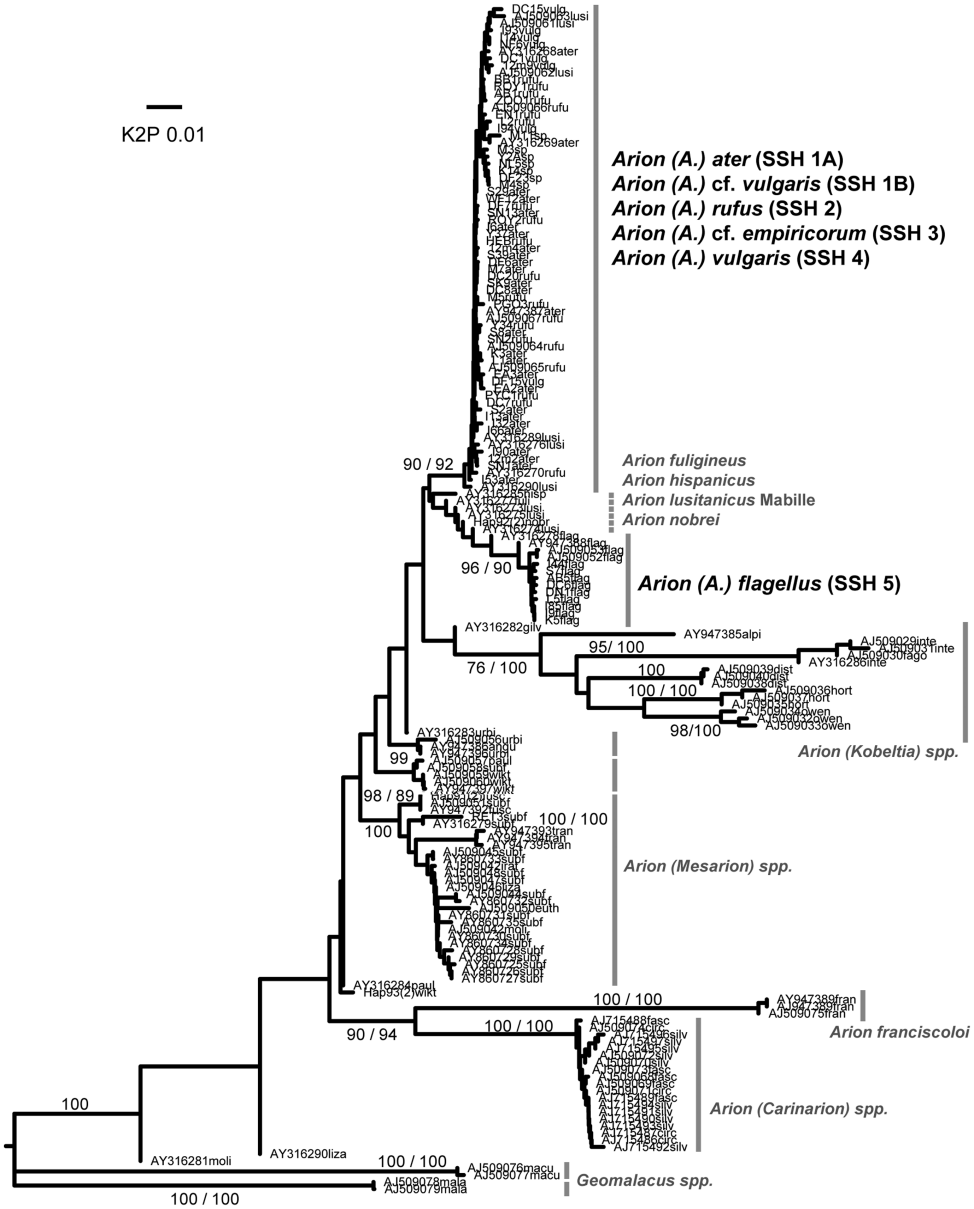
Arionidae. Midpoint-rooted BI tree based on ITS-1 data; values above branches are % bootstrap support (≥75), those below are Bayesian posterior probabilities, expressed as % (≥85).

Each of the 45 putative species was represented by at least two sequences and most comprised several haplotypes and/or sequences from more than one locality (Table 2). Almost all were identically delimited, genetically distinct, and monophyletic (Figs. 1–8; whole family trees available on request). All but two putative species were identically delimited in the whole family and subset analyses. One was PSH 19, *G. maculosus*, in which the Irish and Spanish haplotypes formed a single putative species in the whole family analysis, and two in the subset. The other was in the *Arion (Arion)* dataset, where PSH 1 was delimited in the ABGD analysis of all Arionidae, but split into PSH 1A, PSH 1B, and PSH 1C in the subset.

**Table 2:** Summary of primary species hypotheses (PSH) and secondary species hypotheses (SSH). Haps., number of haplotypes; Inds., number of individuals.

PSH	PSH generated by ABGD		Haps.	Inds.	Mean K2P distances		Monophyly				Morphologically unique?		SSH	Name applied to SSH
					(whole family ABGD analyses)		Whole family		Subset					
	Whole family	Subset			Intraspecific	Interspecific (minimum)	NJ	BI	NJ	BI	External	Internal		
1	(1A + 1B + 1C form a single PSH)	-	23	46	0.02	0.08	66	0	0	0		X	(Split PSH 1)	(no SSH)
1A	“	X	20	36	n/a	n/a	99	100	100	99	X	X	1A	*Arion (Arion) ater* (Linnaeus, 1758)
1B	“	X	2	8	n/a	n/a	93	63	93	59		X	1B+1C	*Arion (Arion)* cf. *vulgaris* Moquin-Tandon, 1855
1C	“	X	1	2	n/a	n/a	n/a	n/a	n/a	n/a	?	?	“	“
2	X	X	9	22	0.00	0.07	100	100	100	100		X	2	*Arion (Arion) rufus* (Linnaeus, 1758)
3	X	X	7	8	0.01	0.07	99	100	100	100		“	3	*Arion (Arion)* cf. *empiricorum* A. Férussac, 1819
4	X	X	14	26	0.00	0.11	100	100	100	100	X	X	4	*Arion (Arion) vulgaris* Moquin-Tandon, 1855
5	X	X	3	24	0.00	0.10	100	100	100	100	X	X	5	*Arion (Arion) flagellus* Collinge, 1893
6	X	X	51	54	0.01	0.17	100	100	100	100	?	X	6	*Arion (Mesarion) fuscus* (O. F. Müller, 1774)
7	X	X	21	47	0.01	0.10	96	100	95	100	X	X	7	*Arion (Mesarion) subfuscus* Draparnaud, 1805 (two, currently indistinguishable evolutionary species [25])
8	X	X	44	45	0.01	0.14	100	100	100	100	“	“	8	“
9	X	X	2	2	0.02	0.13	100	100	100	100	X		9+10	*Arion (Mesarion)* cf. *iratii* Garrido, Castillejo & Iglesias, 1995
10	X	X	2	2	0.03	0.17	100	100	100	100	“		“	“
11	X	X	48	76	0.04	0.20	100	100	100	90	X	X	11	*Arion (Carinarion) circumscriptus* Johnston, 1828 (including *A. (C.) silvaticus* Lohmander, 1937)
12	X	X	13	20	0.01	0.20	100	100	100	100	X	X	12	*Arion (Carinarion) fasciatus* (Nilsson, 1823)
13	X	X	8	22	0.01	0.22	100	100	100	100	X	X	13	*Arion (Kobeltia) distinctus* J. Mabille, 1868
14	X	X	4	10	0.00	0.18	100	100	100	100	X	X	14	*Arion (Kobeltia) hortensis* A. Férussac, 1819
15	X	X	7	22	0.01	0.20	100	100	100	100	X	X	15	*Arion (Kobeltia) intermedius* Normand, 1852
16	X	X	1	3	0.01	0.13	100	97	100	98	?	X	16	*Arion (Kobeltia) occultus* Anderson, 2004
17	X	X	3	12	0.00	0.13	100	100	100	100	X	X	17	*Arion (Kobeltia) owenii* Davies, 1979
18	X	X	4	4	0.00	0.14	100	100	100	100	?	X	18	*Arion (Kobeltia)* cf. *fagophilus* de Winter, 1986
19	X	-	1	4	0.00	0.07	n/a	n/a	n/a	n/a	X	X	19	*Geomalacus maculosus* Allman, 1843
20	X	X	23	25	0.01	0.06	99	100	99	100	X	X	20	*Limax cinereoniger* Wolf, 1803
21	X	X	21	24	0.00	0.06	99	100	99	100	X	X	21	*Limax maximus* Linnaeus, 1758
22	X	X	10	10	0.03	0.08	99	100	99	100	X	X	22	*Limax* cf. *dacampi* Menegazzi, 1854
23	X	X	9	9	0.00	0.11	100	100	100	100	X	X	23	*Limacus flavus* (Linnaeus, 1758)
24	X	X	14	14	0.01	0.11	100	100	100	100		X	24	*Limacus maculatus* (Kaleniczenko, 1851)
25	X	X	26	26	0.01	0.14	100	100	100	100	X	X	25	*Lehmannia marginata* (O. F. Müller, 1774)
26	X	X	8	9	0.01	0.08	100	100	100	100		X	26	*Ambigolimax valentianus* (A. Férussac, 1822)
27	X	X	8	8	0.02	0.08	83	99	97	91		X	27	*Ambigolimax nyctelius* (Bourguignat, 1861)
28	X	X	5	5	0.01	0.13	100	100	100	100	X	X	28	*Malacolimax tenellus* (O. F. Müller, 1774)
29	X	n/a	8	8	0.02	0.05	99	100	n/a	n/a		X	29	*Deroceras agreste* (Linnaeus, 1758)
30	X	n/a	11	14	0.00	0.05	99	84	n/a	n/a		X	30	*Deroceras reticulatum* (O. F. Müller, 1774)
31	X	n/a	21	26	0.02	0.09	99	100	n/a	n/a		X	31	*Deroceras invadens* Reise et al., 2011
32	X	n/a	9	11	0.01	0.08	99	100	n/a	n/a		X	32	*Deroceras panormitanum* (Lessona & Pollonera, 1882)
33	X	n/a	7	10	0.01	0.05	99	100	n/a	n/a	X		33+34+35+36	*Deroceras laeve* (O. F. Müller, 1774) (multiple lineages; further study required)
34	X	n/a	4	5	0.00	0.06	99	100	n/a	n/a	“		“	“
35	X	n/a	4	4	0.01	0.05	99	100	n/a	n/a	“		“	“
36	X	n/a	12	20	0.02	0.05	57	0	n/a	n/a	“		“	“
37	X	n/a	4	4	0.01	0.11	100	100	n/a	n/a	X	X	37	*Milax gagates* (Draparnaud, 1801)
38	X	n/a	10	11	0.00	0.10	100	91	n/a	n/a	X		38	*Tandonia budapestensis* (Hazay, 1881)
39	X	n/a	3	3	0.04	0.11	99	97	n/a	n/a	X		39	*Tandonia* cf. *cristata* (Kaleniczenko, 1851)
40	X	n/a	10	10	0.03	0.10	98	100	n/a	n/a	X	X	40	*Tandonia sowerbyi* (A. Férussac, 1823) (may include *T. marinellii* Liberto et al., 2012)
41	X	n/a	1	2	0.00	0.19	n/a	n/a	n/a	n/a	X	X	41	*Tandonia rustica* (Millet, 1843)
42	X	n/a	5	7	0.01	0.22	100	100	n/a	n/a	X	X	42	*Testacella haliotidea* Draparnaud, 1801
43	X	n/a	5	8	0.00	0.22	100	100	n/a	n/a	X	X	43	*Testacella maugei* (A. Férussac, 1819)
44	X	n/a	2	2	0.00	0.20	100	100	n/a	n/a	X	X	44	*Testacella scutulum* G. B. Sowerby I, 1821
45	X	n/a	5	5	0.00	0.20	100	100	n/a	n/a	X	X	45	*Testacella* cf. *scutulum* G. B. Sowerby I, 1821
n/a	n/a	n/a	2	4	0.00	n/a	n/a	n/a	n/a	n/a	X	X	(46)	*Boettgerilla pallens* Simroth, 1912
n/a	n/a	n/a	2	6	0.00	n/a	n/a	n/a	n/a	n/a	X	X	(47)	*Selenochlamys ysbryda* Rowson & Symondson, 2008

All putative species were genetically highly distinct. Where more than one haplotype was found the minimum mean interspecific distance was always 2.5 or more times greater, often tens of times greater, than the mean intraspecific distance. The intraspecific distance was lower than the prior value given by ABGD in all cases except two putative species in the Milacidae (PSH 39 and PSH 40) in which it slightly exceeded it (Table 2). This may be a result of the limited data available on this family, whose ABGD analysis was also unique in offering an alternative, yet larger, stable number of putative species. Under this alternative PSH, for example, Italian sequences including *Tandonia marinellii* Liberto et al. [56] would be separated from *T. sowerbyi* (Férussac). However, our criterion favours more the conservative PSH.

Almost all putative species with more than one haplotype were strongly supported as monophyletic in all NJ and BI analyses. Again the exception was PSH 1: there was very weak support for the monophyly of PSH 1 as a whole, yet support for the monophyly of PSH 1A and a clade comprising PSH 1B + PSH 1C.

Most (35) of the putative species were morphologically unique, externally and/or (in adult or near-adult) specimens, internally. Most of the remainder were in Arionidae: PSH 1, whose constituents PSH 1A and PSH 1B were each unique; PSH 1C, represented by a single GenBank haplotype from the USA (and discussed because of its delimitation as part of PSH 1); PSH 3, represented in Britain only by a juvenile; PSH 7 and PSH 8, which both corresponded to *A. (M.) subfuscus* (Draparnaud) but could not be satisfactorily distinguished from one another even using the genital features of [28]; and PSH 9 and PSH 10 which both corresponded to *A. (M.) iratii* Garrido et al. but could not be satisfactorily distinguished from one another. In Agriolimacidae, the four PSHs into which *Deroceras laeve* (Müller) was split could not be satisfactorily distinguished, partly because most specimens were aphallic. More detailed discussion of morphological features, geographical distribution, and identification of certain putative species is given below.

In consequence, 36 of the 45 putative species in the PSH were readily accepted as species in the SSH we propose. One of the remainder, PSH 1, was split into two, SSH 1A and SSH 1B + 1C, because of its lack of monophyly and morphological heterogeneity. PSH 7 and PSH 8, although morphologically indistinguishable, were maintained as separate because together they were not monophyletic in any analysis. In contrast, and to remain conservative, two sets of other morphologically indistinguishable putative species were combined (PSH 9 + PSH 10, and PSH 33 + PSH 34 + PSH 35 + PSH 36). The SSH we propose thus recognises a total of 42 species, or 44 including Boettgerillidae and Trigonochlamydidae.

### Comparison of the SSH to the known fauna

Of the 44 SSH species, 32 included one or more GenBank sequence. The remaining 12 had presumably not previously been sequenced for the gene region in question. As expected, many species in each category (24 in the former and 9 in the latter, totalling 33) could readily be considered equivalent to known species in the British and Irish fauna [1] (Table 2, and names on Figs. 1–8). This was consistent with the morphological features we used to identify specimens and, in general, the names of previously identified GenBank sequences. For example, PSH 4 consists of sequences of *A. (A.) vulgaris* (or “*A. lusitanicus*” non Mabille) from six continental countries, and from specimens from around Britain and Ireland whose morphology conforms to that species (e.g. [21], [42]). This confirms it is widespread in Britain and Ireland, including in SW England where it has been recorded since at least the 1960s [2], [21]). Most of the remaining 32 British and Irish SSH species correspond to other widespread and relatively well-characterised species. Two species described from Ireland, *G. maculosus* and *A. (A.) flagellus*, are each moderately closely related to Spanish haplotypes (Fig. 1). We note however that unless the Irish haplotypes are detected in Spain, neither native status or ancient introduction can be ruled out (e.g. see [57], [58]). For brevity, we do not discuss the currently known SSH species further, except for those which appear to show evidence of hybridisation (see below).

This leaves 11 SSH species needing further discussion. In one case, a single SSH species corresponded to more than one known species: *A. (Carinarion) circumscriptus* Johnston and *A. (C.) silvaticus* Lohmander. Together these formed a single putative species, PSH 11 which was monophyletic (Fig. 3). Both names were thus associated with the single species SSH 11, although within it, the British and Irish haplotypes identified as *A. (C.) circumscriptus* and *A. (C.) silvaticus* clustered in separate monophyletic groups. This is consistent with the findings of Geenen et al. [27] who suggested the three widespread *Carinarion* taxa be considered a single biological species. However, like them we found that the third of these, *A. (C.) fasciatus* (Nilsson) (SSH 12) was considerably more distinct than the others and more reliably identifiable morphologically. Given this and the evidence of habitat separation between *A. (C.) circumscriptus* and *A. (C.) silvaticus* in Britain and Ireland [2] we suggest these two be treated as a single species, perhaps with two recognised subspecies. As *A. (C.) fasciatus* is more distinct and formed a separate PSH we retain it as a species.

In another case, two morphologically indistinguishable SSH species corresponded to a single known species. SSH 7 and SSH 8 each corresponded to specimens identified as *A. (M.) subfuscus*, it being uncertain to which (if either) the name should be preferentially applied [26]. Indeed Pinceel and others [25], [26], [28] found this taxon to consist of multiple deeply divergent 16S lineages which they attributed to allopatric divergence and an accelerated rate of mutation. This included two present in Britain and Ireland (their S1 and S2 [25]) that correspond to our SSH 7 and SSH 8. As there was some evidence of interbreeding between these they treated them as evolutionary species requiring further revision before being named, as was possible with *A. (M.) transsylvanus* Simroth, endemic to Romania and Poland [28]. We found limited agreement between SSH 7 and SSH 8 and the subtle morphological characters of S1 and S2 offered by [28]. All S2 specimens had genitalia corresponding to these in that the epiphallus joined the atrium between the bursa and oviduct, but this pattern was also seen in some S1 specimens. S2 was found only in northern, western and upland areas (Table S1) although has also been found sympatric with S1 in central and southeast England by [25]. Until further data are available we treat both as part of the still-enigmatic taxon *A. (M.) subfuscus*.

This left eight SSH species that did not correspond to any of the 36 species in [1], 35 of which were themselves successfully delimited using the same criteria. This represents an increase of 22% on the known fauna, a striking and unexpected finding that led to further investigation of these “additional” species.

### Additional species

Of the eight additional SSH species, four were in Arionidae and one each in Limacidae, Agriolimacidae, Milacidae and Testacellidae. Up to four of the species appear previously undescribed, but we refrain from formal description here until further data are available. We provide brief diagnoses and use the term “cf.” (confer) to indicate a nominal species to which the species should be compared.

We note that none of the eight additional species are morphologically cryptic, at least not by the strictest of definitions, i.e., they are not impossible to reliably identify based on morphology alone [54]. They could therefore have been recognised without sequence data and so can be considered previously overlooked. We also note that six of them were found at more than one widely separated site, with two occurring in both Britain and Ireland. The introduced or native status of each remains open to question. The remaining two, as yet known only from single established populations, are almost certainly accidental introductions.

#### SSH 1B + 1C: *Arion (Arion)* cf. *vulgaris* Moquin-Tandon, 1855

**Figure 9:**
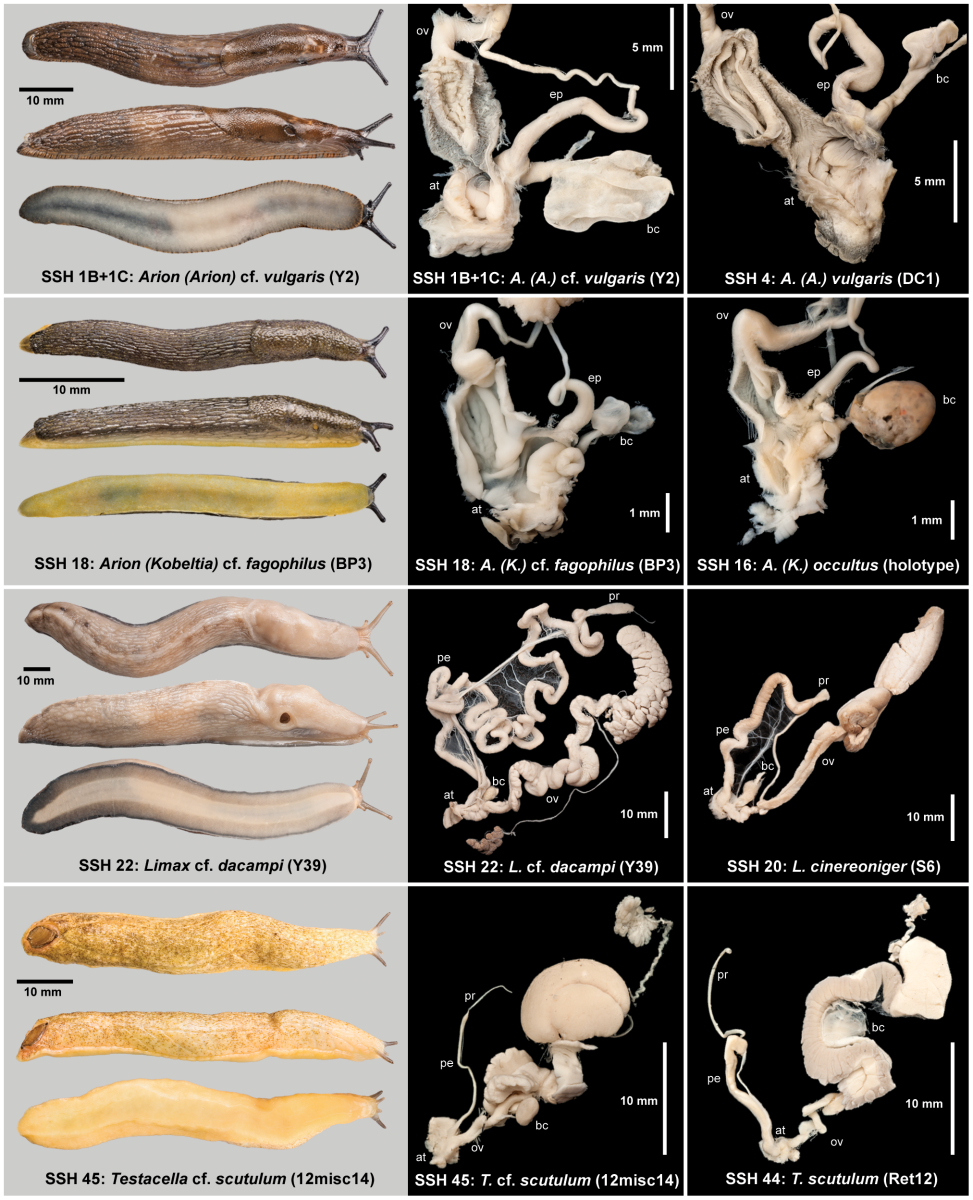
Morphology of four potentially new species. External appearance and salient parts of genitalia shown alongside those from sequenced similar species for comparison. Abbreviations: at, atrium; bc, bursa copulatrix; ep, epiphallus; ov, oviduct; pe, penis; pr, penial retractor muscle.

Potentially a new species. Each of the SSH species in *Arion* subgenus *(Arion)* (with the exception of *A. (A.) flagellus*) was approximately equally genetically distant from one another. SSH 1B was found at four sites in the east of England, from Yorkshire to Kent, with another haplotype near London (a museum specimen collected in 2004). They were mainly from disturbed habitats. Externally the animal is variable and readily confused with either *A. (A.) vulgaris* (SSH 4) or *A .(A.) rufus* (SSH 2). It is generally greyish brown, with faint lateral colour bands persisting in some adults, and the sole is often paler than typical *A. (A.) vulgaris*. It lacks the rocking response of *A. (A.) ater* (SSH 1A) and *A. (A.) rufus* (e.g. [12]). Internally it is very distinct from both *A. (A.) ater* and *A. (A.) rufus*, having an elongate corrugated ligula in the oviduct, generally like that of *A. (A.) vulgaris*. However, the ligula is delicately fringed for all or part of its length as well as being corrugated (Fig. 9). These delicate fringes were not found in the sequenced *A. (A.) vulgaris* specimens. The correct name for this species is difficult to establish at present (see also SSH 3, and the section on hybridisation below) and more data on whether other populations can be consistently distinguished from *A. (A.) vulgaris* is desirable. It appears not to correspond with any of the four species discussed in the arionid review by Jordaens et al. [29] whose “non-Iberian *A. lusitanicus*” presumably corresponds to *A.(A.) vulgaris*. Neither ITS-1 nor 16S data suggest a close relationship between our species and *A. (A.) flagellus,* or to the true Portuguese *A. lusitanicus* Mabille (Fig. 8). It is possible that some of the British “*A. lusitanicus*” populations studied by Davies [21] in her initial discrimination of these from *A. (A.) flagellus* may belong to this species, especially given inconsistencies in the mating behaviour recently highlighted by Dreijers et al. [45]. However, our data suggest that *A. (A.) vulgaris* itself (SSH 4), being much more widespread in Britain and Ireland, is also likely to have been present in Surrey and the other areas discussed by Davies [21]. It is unknown where else in Europe our species occurs and whether it is native to Britain, there being a lack of data from central and southern France in particular. Noble & Jones [17] discussed a “non-pest” form of *A. (A.) vulgaris* (again as “*A. lusitanicus*”) from southern France while Noble [42] discussed alternative Pyrenean forms that might correspond to this species. The most similar available 16S sequences are those recognised in some analyses as PSH 1C (Fig. 1). These are from “*Arion rufus*” from the west coast of the USA (Washington State: [30]), whose morphology may deserve further investigation.

#### SSH 3: *Arion (Arion)* cf. *empiricorum* A. Férussac 1819

Sequences of this SSH species have been identified as “*Arion rufus*” from Belgium, Denmark, and (as introduced) Canada and the USA. In Britain it was represented by a single juvenile from a cemetery in central London identified as (and sympatric with) *A.(A.) vulgaris*, whose juveniles can look very similar. It has been proposed [59] that the name *A. (A.) rufus* Linnaeus be applied to the species occurring in Britain (presumably the widespread and common SSH 2 in our study). Alternative names available for similar continental species include *Arion empiricorum* Férussac, [1], but this cannot be resolved here. The London population is likely to be an introduction and as with SSH1B + 1C, should be considered a potential plant pest.

#### SSH 9 + 10: *Arion (Mesarion)* cf. *iratii* Garrido, Castillejo & Iglesias, 1995

This SSH combines PSH 9 and PSH 10, both from upland forestry areas in the Brecon Beacons National Park and South Wales valleys. They could not be distinguished morphologically but both differ from all sampled British and Irish *A. (M.) subfuscus* and *A. (M.) fuscus* in having dark spots or speckles on the back in at least some individuals. This feature is shared with three species described by Garrido et al., 1995 [60] from the Pyrenees which differ from each other only very subtly: *A. (M.) iratii*, *A. (M.) lizarrusti*, and *A. (M.) molinae.* Given the accelerated rate of 16S evolution posited for *Mesarion* species [25] it is not clear whether the constituent PSHs should be combined in one SSH or not, but analyses suggest both are related to *A. (M.) iratii* and *A. (M.) lizarrusti* rather than to *A. (M.) subfuscus, A. (M.) fuscus*, *A. (M.) transsylvanus* etc. (Fig. 2). Neither Wales nor the Pyrenees were represented in the analysis of Pinceel et al. [28], [29]; indeed they noted that sampling in Iberia had yielded none of the *A. (M.) subfuscus* lineages S1–S5. This SSH is therefore provisionally associated with *A. (M.) iratii*. Its introduction to Britain with forestry or industry cannot be ruled out, but neither can a native distribution that includes the highest uplands in southern Britain.

#### SSH 18: Arion (Kobeltia) cf. fagophilus (de Winter, 1986)

Potentially a new species. Genetically it is distinct from all British and Irish *Kobeltia* and from the central European *A. (K.) obesoductus* Reischutz (formerly *A. (K.) alpinus* Pollonera) (Table 2, Fig. 3). Morphologically it is most similar to *A. (K.) occultus* Anderson from Northern Ireland and *A. (K.) fagophilus* de Winter from the western Pyrenees. Externally, the coarser tubercles distinguish the new species from all British or Irish *Kobeltia* except *A. (K.) occultus*. The latter is yellower and flatter, with even coarser tubercles. Both resemble *A. (K.) fagophilus* externally except in tentacle colour, which is bright red in *A. (K.) fagophilus* [61] versus cold blue-black in these species. Davies [20] considered tentacle colour one of the most reliable external features distinguishing *Kobeltia* species. Internally, this species lacks an epiphallus process and differs from *A. (K.) fagophilus* and *A. (K.) occultus* in having a donut-like swelling at the entrance of the bursa copulatrix to the atrium, rather than a thickening as in the other species (Fig. 9) [23], [61]. It is common at several sites in lowland river valleys in South Wales, in wet *Alnus* woodland. It is probably an introduction from the Pyrenees, although this small and indistinct species could be an overlooked native restricted to this relatively natural habitat type.

#### SSH 22: *Limax* cf. *dacampi* Menegazzi, 1854

Potentially a new species. Genetically it is clearly not part of the two known British and Irish species which form separate PSHs (Table 2, Fig. 4). It forms a clade (Fig. 4) with sequences from the Apennines of central Italy to which no species name is applied, and another group comprising sequences from Italy, Switzerland and Croatia that depositors identified as *Limax* cf. *dacampi* Menegazzi [32]. In the whole family ABGD analysis, both clades form a single PSH but form two PSHs in the analysis of *Limax* only. In Britain it was found at a single site in North-east Yorkshire, in an ornamental mixed woodland in the grounds of a school. Adults and juveniles have been found there on several occasions over the past two years (A. Norris & T. Crawford, pers. comm.). The species is externally and internally distinct from the other British species (e.g. as monographed by Quick [12]), notably in having a much longer penis than either (both in absolute terms and in being longer than the body even when retracted) (Fig. 9). It is almost certainly an introduction from central Italy.

#### SSH 32: *Deroceras panormitanum* (Lessona & Pollonera, 1882)

An established population in a public city garden in South Wales has the internal anatomy of this Sicilian/Maltese species (one blunt penial appendage, one tapering) and an almost identical COI haplotype (Fig. 6). This species and its relatives were recently thoroughly revised based on molecular, morphological and behavioural data [33]. Its external morphological variation overlaps with that of the widely introduced *D. invadens* Reise et al., so it may have been overlooked elsewhere. In Britain, it is probably a recent introduction.

#### SSH 39: *Tandonia cf. cristata* (Kaleniczenko, 1851)

Found in South Wales and western Ireland in allotments, adjacent woodlands and a churchyard. Museum collections suggest that it is more widespread than our data indicate (although we could not amplify DNA from these). It has evidently been misidentified as juvenile *T. budapestensis* (Hazay), *T. sowerbyi* or *Milax gagates* (Draparnaud) in the past but is genetically distinct from these (Fig. 7). It differs from these in its smaller size, generally paler colour, plain sole, and in having dark pigment confined to the spaces between tubercles. Using Wiktor's key [62] it keys to *Tandonia cristata* (Kaleniczenko), a species of countries bordering the Black Sea. There is some discrepancy between accounts of the internal anatomy of this species [62], [63], and there are many similar species. Until these can be better studied we consider the British and Irish species likely to represent *T. cristata*, which would be introduced. It is potentially a root crop pest like other *Tandonia* species.

#### SSH 45: *Testacella cf. scutulum* G. B. Sowerby I, 1821

Potentially a new species. Genetically it is as distinct from *T. scutulum* Sowerby as the other two *Testacella* species are from one another (Fig. 7), and forms a PSH separate to them in delimitation analysis (Table 2). Morphologically, it is close only to *T. scutulum* with which it keys out in the synoptic key of Nardi & Bodon [64]. However, the two differ in the width of the penis (apparently independently of the size of the animal) and in whether or not the penis tapers (Fig. 9). Externally, they differ in body colour and in the shape formed by the meeting of the two dorsal grooves, although these features might not be consistent. The subterranean Testacellidae are rarely collected so only limited material is available and intraspecific morphological variation remains poorly understood [65] (although there is abundant evidence that *T. haliotidea* Draparnaud and *T. scutulum* are distinct, contrary to [2]). It also remains to be established to which of the two similar species Sowerby's name ought to be restricted. Early descriptions of the anatomy of *T. scutulum* from near the type locality in London [11], [66] show a broad, tapering penis. Both species are probably introductions from the Mediterranean [2]; recent figures suggest both may occur in Italy [67].

### Hybridisation

Here, intraspecific hybridisation is considered the exchange of genes between PSHs that would otherwise be considered phylogenetic species, and would otherwise correspond precisely to morphologically distinct and widely-recognised species (e.g. “*A.(A.) vulgaris*”). Although limited, our data suggest that hybridisation may have occurred in the larger Arionidae. More unexpectedly, contrasts between mtDNA and morphology also suggest hybridisation in Agriolimacidae and Limacidae.

Interspecific hybridisation has long been suspected, if not fully proven, among the large Arionidae, in particular between *A. (A.) ater* and *A. (A.) rufus* and between one or both of these and *A. (A.) vulgaris* (e.g. [15], [42], [43], [44], [68], [69]). Recently, Dreijers et al. [45] showed experimentally that German *A. (A.) vulgaris* (as *A. lusitanicus*) and German *A. (A.) rufus* can reciprocally exchange sperm. ITS-1 sequences showed far less variability than mtDNA sequences. NJ and BI analyses show that all five SSHs in the larger Arionidae (SSHs 1A, 1B, 2, 3 and 4) form a single clade without internal structure, to the exclusion of *A. (A.) flagellus* (SSH 5) and all other Arionidae (Fig. 8). Similar conflicts between mtDNA and ITS-1 data have been interpreted as evidence of hybridisation or a shared nuclear gene pool in arionid studies [25], [27]. By these criteria, hybridisation might well have occurred between any combination of the five SSHs in the larger clade, either before or since their arrival in Britain or Ireland. In contrast, *A. (A.) flagellus* is genetically distinct from any of them in both mtDNA and ITS-1. We conclude that evidence for its hybridisation with other British or Irish species is lacking.

Morphologically, we found that all adult specimens we sequenced could be referred to one of the known British species in the large Arionidae, or the newly-discovered SSH 1B, and were unconvinced that any individual was indisputably hybrid. We found no specimens whose species assignment by morphology was incompatible with their assignment by mtDNA (unlike the non-arionid examples below). It has been suggested that hybrids are morphologically recognisable [17], [42], [68] but this was not clarified by our data. It remains possible that hybrids in this group (especially after the F1 generation) could resemble either parent species and be effectively impossible to distinguish from them morphologically [46]. Elsewhere in Europe, such hybrids might account for the inclusion of GenBank haplotypes of Belgian, Polish, Spanish and Czech “*A. rufus*”, and Polish “*A. lusitanicus*” in our PSH 1A. We nonetheless remain confident that this SSH be called *A. (A.) ater*, it being the only SSH that included jet-black individuals from remote western and upland regions, considered typical of *A. (A.) ater* [11], [17], [42], [68] (Table S1). The identities of the other SSHs in the group are discussed above. While the taxonomy may be revised in future, we provisionally recognise five species in this group in the British fauna, three of which occur in Ireland (not including *A. (A.) flagellus*).

In Agriolimacidae, in ABGD, NJ and BI analyses (Fig. 6) *Deroceras reticulatum* (Müller) and *D. agreste* (Linnaeus) formed two PSHs/clades. Using the internal morphological criteria of Wiktor [70] however, neither morphospecies was monophyletic and so they cannot be accepted as separate SSHs here unless some individuals are considered hybrids. All individuals with *D. agreste* anatomy had a *D. agreste* mtDNA sequence, but some individuals with *D. reticulatum* anatomy had a *D. agreste* mtDNA sequence. These were largely from the same sites as the *D. agreste* individuals, or nearby. The pattern could be explained by one-way introgression of *D. reticulatum* genes into *D. agreste* populations. As mtDNA is maternally inherited in pulmonates, the *D. reticulatum* individuals in the *D. agreste* clade would be introgressed hybrids descended from a *D. agreste* mother whose eggs were fertilised by *D. reticulatum* sperm. That no *D. agreste* individuals with *D. reticulatum* mtDNA were found may indicate that fertilisation in the opposite direction has not occurred, is rarer, or results in sterile offspring. One-way interspecific sperm transfer is known in other *Deroceras* species [33]. In Britain and Ireland *D. agreste* is a rare, northerly distributed post-glacial relict species characteristic of uplands and other wild habitats, while *D. reticulatum* is a common, widespread and often synanthropic species [2], [13]. If the speculation is correct, this interaction between the species could partly explain their habitat and range discrepancies, with the post-glacial *D. agreste* in long-term retreat exacerbated by hybridisation with *D. reticulatum*.

Similarly in Limacidae, in ABGD, NJ and BI analyses (Fig. 5) *Limacus flavus* (Linnaeus) and *L. maculatus* (Kaleniczenko) formed two PSHs/clades. Again, using internal morphological criteria [71] neither morphospecies was monophyletic.The only morphologically typical adult *L. flavus* (i.e. one with the bursa copulatrix duct originating high up on the vagina; [71]) was in PSH 23 in which all other specimens either had immature genitalia or were typical of *L. maculatus* (i.e. with the bursa duct obtaining from the base of the vagina, atrium, or base of the penis). Many of them resembled *L. maculatus* externally [13], [14]. All specimens in PSH 24 resembled *L. maculatus* internally and externally, including one from near the type locality in Crimea, Ukraine. GenBank sequences attributed to *L. flavus* were split between the two clades, with continental European sequences [31] in the *L. flavus* clade and British sequences in the *L. maculatus* clade. The pattern can be interpreted in a similar way to *D. agreste/reticulatum*. Neither *Limacus* species is considered native although *L. flavus* has been known in Britain since at least 1685 [2], [8] and was the only *Limacus* known in Ireland in 1891 [10]. Anecdotal evidence and the increasing frequency of *L. maculatus* being recorded in Britain and Ireland (e.g. [72], also personal observations) suggest it is becoming common and widespread while *L. flavus* becomes rarer. One-way introgression from the more recent invader could explain this trend.

## Conclusion

This study demonstrates how a simple, geographically extensive yet morphologically informed approach to DNA sampling can be effective in screening a fauna for additional taxa and other changes resulting from past invasions. The detection of so many additional species, and the evidence for hybridisation in Agriolimacidae and Limacidae, were unexpected. The proportion (22%) of additional species revealed is remarkably high compared to other recent studies and given the long history of study of this fauna. For example, no additional taxa were found in DNA barcoding surveys of the complete Irish solitary bee fauna of 55 species [73] or the Welsh native and archaeophyte angiosperm and conifer flora of over 1000 species [74]. Our estimates also exceed the proportion of cases of deep intraspecific divergence (i.e. potential additional species) found and discussed among Bavarian myriapods (3% of 122 species [75]), Romanian butterflies (4% of 180 species [76]) or Bavarian geometrid moths (5% of 400 species [77]). With the exception of [75] these studies were largely based on existing museum collections so may have overlooked additional taxa in the field, but as we found such taxa may also be present in existing collections as in the case of *Tandonia cf. cristata*. Only among British and Irish earthworms [78] and Scottish tardigrades [79] have similar studies reported as high a proportion of additional putative taxa. These are groups with a much less rich history of taxonomic description, participatory study and recording than slugs, and do not include as many potentially serious pests. Indeed, further undetected slug species may already be lurking, particularly in large urban areas which our survey could not thoroughly cover. We conclude that although the British and Irish slug fauna is well-studied, it was far from fully-known, even as recently as 2008 [1]. If further invasions are to be detected or controlled, this and other slug faunas worldwide will need to be watched more closely in future.

## Supporting Information

Figure S1 **ABGD analysis for Arionidae.** Arrow indicates selected PSH and its corresponding position on the distribution of pairwise K2P distances.
(TIF)

Figure S2 **ABGD analysis for Limacidae and Agriolimacidae.** Arrow indicates selected PSH and its corresponding position on the distribution of pairwise K2P distances.
(TIF)

Figure S3 **ABGD analysis for Milacidae and Testacellidae.** Arrow indicates selected PSH and its corresponding position on the distribution of pairwise K2P distances.
(TIF)

Table S1 **Specimen data, haplotypes, and GenBank accession numbers.**
(XLS)

Table S2 **GenBank data and haplotypes included in analyses.**
(XLS)

References S1 **Additional references cited in Table S2.**
(DOC)

## References

[1] Anderson R (2008) Annotated list of the non-marine Mollusca of Britain & Ireland. http://www.conchsoc.org/node/540. Accessed 2013Jan 16.

[2] Kerney M (1999) Atlas of the land and freshwater molluscs of Britain and Ireland. Colchester, UK: Harley Books. 264 p.

[3] Herbert DG (2010) The introduced terrestrial Mollusca of South Africa. South African National Biodiversity Institute Biodiversity Series 15. Pretoria, South Africa: South African National Biodiversity Institute. 108 p.

[4] Barker GM (1999) Naturalised terrestrial Stylommatophora (Mollusca: Gastropoda). Fauna of New Zealand 38. Lincoln, New Zealand: Manaaki, Whenua Press. 254 p.

[5] Grimm FW, Forsyth RG, Schueler FW, Karstad A (2009) Identifying land snails and slugs in Canada: introduced species and native genera. Ottowa, Canada: Canadian Food Inspection Agency. 168 p.

[6] McDonnell RJ, Paine TD, Gormally MJ (2009) Slugs: a guide to the invasive and native fauna of California. University of California publication no. 8336. Oakland, California, USA: University of California. 21 p. http://anrcatalog.ucdavis.edu/Items/8336.aspx.

[7] McDonnell RJ, Rugman-Jones P, Backeljau T, Bruegelmans K, Jordaens K, et al (2011) Molecular identification of the exotic slug *Arion subfuscus* sensu stricto (Gastropoda: Pulmonata) in California, with comments on the source location of introduced populations. Biol Invasions 13: 61–66.

[8] Lister M (1685) Historiae Conchyliorum. London, UK. 556 p.

[9] Linnaeus C (1758) Systema Naturae per Regna Tria Naturae, secundum classes, ordines, genera, species, cum characteribus, differentiis, synonymis, locis. Editio decimal reformata. Stockholm, Sweden: Laurentius Salvius. 824 p.

[10] Scharff RF (1891) The slugs of Ireland. Scientific Transactions of the Royal Dublin Society Series II 4: 4513–563.

[11] Taylor JW (1902–1907) Monograph of the land & freshwater Mollusca of the British Isles 2: Testacellidae, Limacidae, Arionidae. Taylor Bros., Leeds, UK. 312 p.

[12] Quick HE (1960) British slugs (Pulmonata; Testacellidae, Arionidae, Limacidae) Bull Br Mus. 6: 103–226.

[13] Kerney MP, Cameron RAD (1979) A field guide to the land snails of Britain and North-west Europe. London, UK: Collins. 288 p.

[14] Cameron RAD, Jackson N, Eversham B (1983) A field key to the slugs of the British Isles. Field Studies Journal 5: 807–824.

[15] Burnet B (1972) Enzyme protein polymorphism in the slug *Arion ater* . Genet Res 20: 161–173.

[16] Foltz DW, Ochman H, Selander RK (1984) Genetic diversity and breeding systems in terrestrial slugs of the families Limacidae and Arionidae. Malacologia 25: 593–605.

[17] Noble LR, Jones CS (1996) A molecular and ecological investigation of the large arionid slugs of North-West Europe: the potential for new pests. In: Symondson WOC, Liddell JE, editors The ecology of agricultural pests: Biochemical approaches. Systematics Association Special Volume Series 53. London, UK: Chapman & Hall. pp 93–131.

[18] South A (1992) Terrestrial Slugs: Biology, ecology and control. London, UK: Chapman & Hall. 428 p.

[19] Barker GM (ed.) (2002) Molluscs as crop pests. Wallingford, UK: CABI Publishing. 468 p.

[20] Davies SM (1979) Segregates of the *Arion hortensis* complex (Pulmonata: Arionidae) with the description of a new species, *Arion owenii* . J Conchol 30: 123–128.

[21] Davies SM (1987) *Arion flagellus* Collinge and *A. lusitanicus* Mabille in the British Isles: a morphological, biological and taxonomic investigation. J Conchol 32: 339–354.

[22] Philp EG (1987) *Tandonia rustica* (Millet), a slug new to the British Isles. J Conchol 32: 302–303.

[23] Anderson R (2004) *Arion occultus* sp. nov., a new slug in *Arion* subgenus *Kobeltia*, from Ireland. J Conchol 38: 13–26.

[24] Rowson B, Symondson WOC (2008) *Selenochlamys ysbryda* sp. nov. from Wales, UK: a *Testacella*-like slug new to Western Europe (Stylommatophora: Trigonochlamydidae). J Conchol 39: 537–552.

[25] Pinceel J, Jordaens K, Backeljau T (2005) Extreme mtDNA divergences in a terrestrial slug (Gastropoda, Pulmonata, Arionidae): accelerated evolution, allopatric divergence and secondary contact. J Evol Biol 18: 1264–1280. 16135122 10.1111/j.1420-9101.2005.00932.x

[26] Pinceel J, Jordaens K, Van Houtte N, de Winter AJ, Backeljau T (2004) Molecular and morphological data reveal cryptic taxonomic diversity in the terrestrial slug complex *Arion subfuscus/fuscus* (Mollusca, Pulmonata, Arionidae) in continental north-west Europe. Biol J Linn Soc Lond 83: 23–38.

[27] Geenen S, Jordaens K, Backeljau T (2006) Molecular systematics of the *Carinarion* complex (Mollusca: Gastropoda: Pulmonata) : a taxonomic riddle caused by a mixed breeding system. Biol J Linn Soc Lond 89: 589–604.

[28] Jordaens K, Pinceel J, Van Houtte N, Breugelmans K, Backeljau T (2010) *Arion transsylvanus* (Mollusca, Pulmonata, Arionidae): rediscovery of a cryptic species. Zool Scr 39: 343–362.

[29] Jordaens K, de Winter A, Bruegelmans K, Pinceel J, Geenen S, et al (2011) Recent advances in the taxonomy of the NW European slugs of the genus *Arion* (Mollusca, Gastropoda, Pulmonata). International Organisation for Biological Control West Palaearctic Regional Section Bulletin 64: 23–39.

[30] Barr NB, Cook A, Elder P, Molongoski J, Prasher D, et al (2009) Application of a DNA barcode using the 16S rRNA gene to diagnose pest *Arion* species in the USA. J Molluscan Stud 75: 187–191.

[31] Nitz B, Heim R, Schneppat UE, Hyman I, Haszprunar G (2009) Towards a new standard in slug species descriptions: the case of *Limax sarnensis* Heim & Nitz n. sp. (Pulmonata: Limacidae) from the western central Alps. J Molluscan Stud 75: 279–224.

[32] Nitz B, Falkner G, Haszprunar G (2010) Inferring multiple Corsican *Limax* (Pulmonata: Limacidae) radiations: a combined approach using morphology and molecules. In: Glaubrecht M, editor: Evolution in action - case studies in adaptive radiation, speciation and the origin of biodiversity. Berlin, Germany: Springer Verlag. pp. 405–435.

[33] Reise H, Hutchinson JMC, Schunack S, Schlitt B (2011) *Deroceras panormitanum* and congeners from Malta and Sicily, with a redescription of the widespread pest slug as *Deroceras invadens* n. sp. Folia Malacol 19: 201–233.

[34] Sites JW, Marshall JC (2004) Operational criteria for delimiting species. Annu Rev Ecol Evol Syst 35: 199–227.

[35] Galtier N, Nabholz B, Glémin S, Hurst GDD (2009) Mitochondrial DNA as a marker of molecular diversity: a reappraisal. Mol Ecol 18: 4541–4550. 19821901 10.1111/j.1365-294X.2009.04380.x

[36] Davison A, Blackie RLE, Scothern GP (2009) DNA barcoding of stylommatophoran land snails: a test of existing sequences. Mol Ecol Resour 9: 1092–1101. 21564847 10.1111/j.1755-0998.2009.02559.x

[37] Prévot V, Jordaens K, Sonet G, Backeljau T (2013) Exploring species level taxonomy and species delimitation methods in the facultatively self-fertilizing land snail genus *Rumina* (Gastropoda: Pulmonata). PLoS ONE 8(4): e60736. 23577154 10.1371/journal.pone.0060736 PMC3618274

[38] Puillandre N, Lambert A, Brouillet S, Achaz G (2012) ABGD, Automatic Barcode Gap Discovery for primary species delimitation. Mol Ecol 21: 1864–1877. 21883587 10.1111/j.1365-294X.2011.05239.x

[39] Puillandre N, Modica MV, Zhang Y, Sirovich L, Boisselier M-C, et al (2012) Large scale species delimitation method for hyperdiverse groups. Mol Ecol 21: 2671–2691. 22494453 10.1111/j.1365-294X.2012.05559.x

[40] Pons J, Barraclough TG, Gomez-Zurita J, Cardoso A, Duran DP, et al (2006) Sequence-based species delimitation for the DNA taxonomy of undescribed insects. Syst Biol 55: 595–609. 16967577 10.1080/10635150600852011

[41] Lim GS, Balke M, Meier R (2011) Determining species boundaries in a world full of rarity: singletons, species delimitation methods. Syst Biol 61: 165–169. 21482553 10.1093/sysbio/syr030

[42] Noble LR (1992) Differentiation of large arionid slugs (Mollusca, Pulmonata) using ligula morphology. Zool Scr 21: 255–263.

[43] Hatteland BA, Noble LR, Schander C, Skage M, Solhøy T (2010) Differentiation of the invasive *Arion lusitanicus* and the native *Arion ater* (Pulmonata, Arionidae) in Norway using morphology and genetics. In: Hatteland BA. 2010. Predation by carabid beetles (Coleoptera, Carabidae) on the invasive Iberian slug *Arion lusitanicus*. PhD thesis, University of Bergen, Norway.

[44] Roth S, Hatteland B, Solhoy T (2012) Some notes on reproductive biology and mating behaviour of *Arion vulgaris* Moquin-Tandon 1855 in Norway including a mating experiment with a hybrid of *Arion rufus* (Linnaeus 1758) x *ater* (Linnaeus 1758). J Conchol 41: 249–257.

[45] Dreijers E, Reise H, Hutchinson JMC (2013) Mating of the slugs *Arion lusitanicus* auct. non Mabille and *A. rufus* (L.); different genitalia and mating behaviours are incomplete barriers to interspecific sperm exchange. J Molluscan Stud 79: 51–63.

[46] Evans NJ (1986) An investigation of the status of the terrestrial slugs *Arion ater ater* (L.) and *Arion ater rufus* (L.) (Mollusca, Gastropoda, Pulmonata) in Britain. Zool Scr 15: 313–322.

[47] Palumbi SR, Benzie J (1991) Large mitochondrial DNA differences between morphologically similar Penaeid shrimps. Mol Mar Biol Biotechnol 1: 27–34. 1669002

[48] Slotsbo S, Hansen LM, Jordaens K, Backeljau T, Malmendal A, et al (2012) Cold tolerance and freeze-induced glucose accumulation in three terrestrial slugs. Comp Biochem Physiol A Mol Integr Physiol 161: 443–449. 22248916 10.1016/j.cbpa.2012.01.002

[49] Folmer O, Black M, Hoeh W, Lutz R, Vrijenhoek R (1994) DNA primers for amplification of mitochondrial cytochrome c oxidase subunit I from diverse metazoan invertebrates. Mol Mar Biol Biotechnol 3: 294–299. 7881515

[50] Hall TA (1999) BioEdit: a user-friendly biological sequence alignment editor and analysis program for Windows 95/98/NT. Nucleic Acids Symp Ser 41: 95–98.

[51] Wade CM, Mordan PB, Naggs F (2006) Evolutionary relationships among the pulmonate land snails and slugs (Pulmonata, Stylommatophora). Biol J Linn Soc Lond 87: 593–610.

[52] Tamura K, Peterson D, Peterson N, Stecher G, Nei M, et al (2011) MEGA5: Molecular Evolutionary Genetics Analysis using Likelihood, Distance, and Parsimony methods. Mol Biol Evol 28: 2731–2739. 21546353 10.1093/molbev/msr121 PMC3203626

[53] Huelsenbeck JP, Ronquist F (2001) MrBAYES: Bayesian inference of phylogenetic trees. Bioinformatics 17: 754–755. 11524383 10.1093/bioinformatics/17.8.754

[54] Bickford D, Lohman DJ, Sodhi NS, Ng PKL, Meier R, et al (2007) Cryptic species as a window on diversity and conservation. Trends Ecol Evol 22: 148–155. 17129636 10.1016/j.tree.2006.11.004

[55] Padial JM, Miralles A, De la Riva I, Vences M (2010) The integrative future of taxonomy. Front Zool 7: 16. 20500846 10.1186/1742-9994-7-16 PMC2890416

[56] Liberto F, Giglio S, Colomba MS, Sparacio I (2012) New and little known land snails from Sicily (Mollusca Gastropoda) Biodivers J. 3: 201–228.

[57] Grindon AJ, Davison A (2013) Irish *Cepaea nemoralis* land snails have a cryptic Franco-Iberian origin that is most easily explained by the movements of Mesolithic humans. PLoS ONE 8: e65792. 23840368 10.1371/journal.pone.0065792 PMC3686809

[58] Valtueña FJ, Preston CD, Kadereit JW (2012) Phylogeography of a Tertiary relict plant, *Meconopsis cambrica* (Papaveraceae), implies the existence of northern refugia for a temperate herb. Mol Ecol 21: 1423–1437. 22320448 10.1111/j.1365-294X.2012.05473.x

[59] van Regteren Altena CO (1963) Notes sur limaces. Basteria 27: 1–6.

[60] Garrido C, Castillejo J, Iglesias J (1995) The *Arion subfuscus* complex in the eastern part of the Iberian Peninsula, with redescription of *Arion subfuscus* (Draparnaud, 1805) (Gastropoda: Pulmonata: Arionidae). Archiv für Molluskenkunde 124: 103–118.

[61] De Winter AJ (1986) Little known and new south-west European slugs (Pulmonata: Agriolimacidae, Arionidae). Zool Meded 60: 135–158.

[62] Wiktor A (1987) Milacidae (Gastropoda, Pulmonata) - systematic monograph. Annal Zool 41: 153–319.

[63] Likharev IM, Wiktor A (1980) The fauna of slugs of the USSR and adjacent countries (Gastropoda terrestria nuda). Fauna SSSR. Mollyuski III (5). Saint Petersburg, Russia. 438 p.

[64] Nardi G, Bodon M (2011) Una nuova specie di *Testacella* Lamarck, 1801, per l'Italia Settentrionale (Gastropoda: Pulmonata: Testacellidae). Boll Malacol 48: 150–164.

[65] De Winter AJ, van Nieulande FAD (2011) *Testacella haliotidea* Draparnaud, 1801 in the Netherlands (Gastropoda Pulmonata, Testacellidae). Basteria 75: 11–22.

[66] Taylor JW (1888) On the specific distinctness and the geographical distribution of *Testacella scutulum* G. B. Sowerby. J Conchol 5: 337–347.

[67] Liberto F, Renda W, Colomba MS, Giglio S, Sparacio I (2011) New records of *Testacella scutulum* Sowerby, 1821 (Gastropoda, Pulmonata, Testacellidae) from Southern Italy and Sicily. Biodivers J 2: 27–34.

[68] Cain AJ, Williamson MH (1958) Variation and specific limits in the *Arion ater* aggregate. Proc Malac Soc Lond 33: 72–86.

[69] Hagnell J, Schander C, von Proschwitz T (2003) Hybridisation in arionids: the rise of a super slug? In: DussartGBJ, editor: Slugs & snails: agricultural, veterinary & environmental perspectives. Symposium Proceedings No. 80. Alton, UK: British Crop Protection Council/Malacological Society of London. pp 221–226.

[70] Wiktor A (2000) Agriolimacidae (Gastropoda: Pulmonata) – a systematic monograph. Annal Zool 49: 347–590.

[71] Wiktor A (2001) Fauna Graeciae 8. The Slugs of Greece (Arionidae, Milacidae, Limacidae, Agriolimacidae – Gastropoda, Stylommatophora). Irakleio, Greece: Natural History Museum of Crete. 241 p.

[72] Habitas (2010) MolluscIreland. Available: http://www.habitas.org.uk/molluscireland/index.html. Accessed 2013 Jan 16.

[73] Magnacca KN, Brown MJ (2012) DNA barcoding a regional fauna: Irish solitary bees. Mol Ecol Resour 12: 990–998. 22931682 10.1111/1755-0998.12001

[74] de Vere N, Rich TCG, Ford CR, Trinder SA, Long C, et al (2012) DNA barcoding the native flowering plants and conifers of Wales. PLoS ONE 7: e37945. 22701588 10.1371/journal.pone.0037945 PMC3368937

[75] Spelda J, Reip HS, Oliveira-Biener U, Melzer RR (2011) Barcoding Fauna Bavarica: Myriapoda - a contribution to DNA sequence-based identifications of centipedes and millipedes (Chilopoda, Diplopoda). Zookeys 156: 123–139. 10.3897/zookeys.156.2176 PMC3253575 22303099

[76] Dincă V, Zakharov EV, Hebert PND, Vila R (2010) Complete DNA barcode reference library for a country's butterfly fauna reveals high performance for temperate Europe. Proc R Soc Lond B Biol Sci 278: 347–355. 10.1098/rspb.2010.1089 PMC3013404 20702462

[77] Hausmann A, Haszprunar G, Hebert PDN (2011) DNA barcoding the geometrid fauna of Bavaria (Lepidoptera): successes, surprises, and questions. PLoS ONE: e17134. 10.1371/journal.pone.0017134 PMC3040642 21423340

[78] King RA, Tibble AL, Symondson WOC (2008) Opening a can of worms: unprecedented sympatric cryptic diversity within British lumbricid earthworms. Mol Ecol 17: 4684–4698. 18992008 10.1111/j.1365-294X.2008.03931.x

[79] Blaxter M, Elsworth B, Daub J (2004) DNA taxonomy of a neglected animal phylum: an unexpected diversity of tardigrades. Proc R Soc Lond B Biol Sci 271: S189–S192. 10.1098/rsbl.2003.0130 PMC1810026 15252980

