# Supplementary figures and images for: The Slugs of Britain and Ireland: Undetected and Undescribed Species Increase a Well-Studied, Economically Important Fauna by More Than 20%
Source: PLoS One. 2014 Apr 16;9(4):e91907. doi: 10.1371/journal.pone.0091907 (PMC3989179; doi:10.1371/journal.pone.0091907)

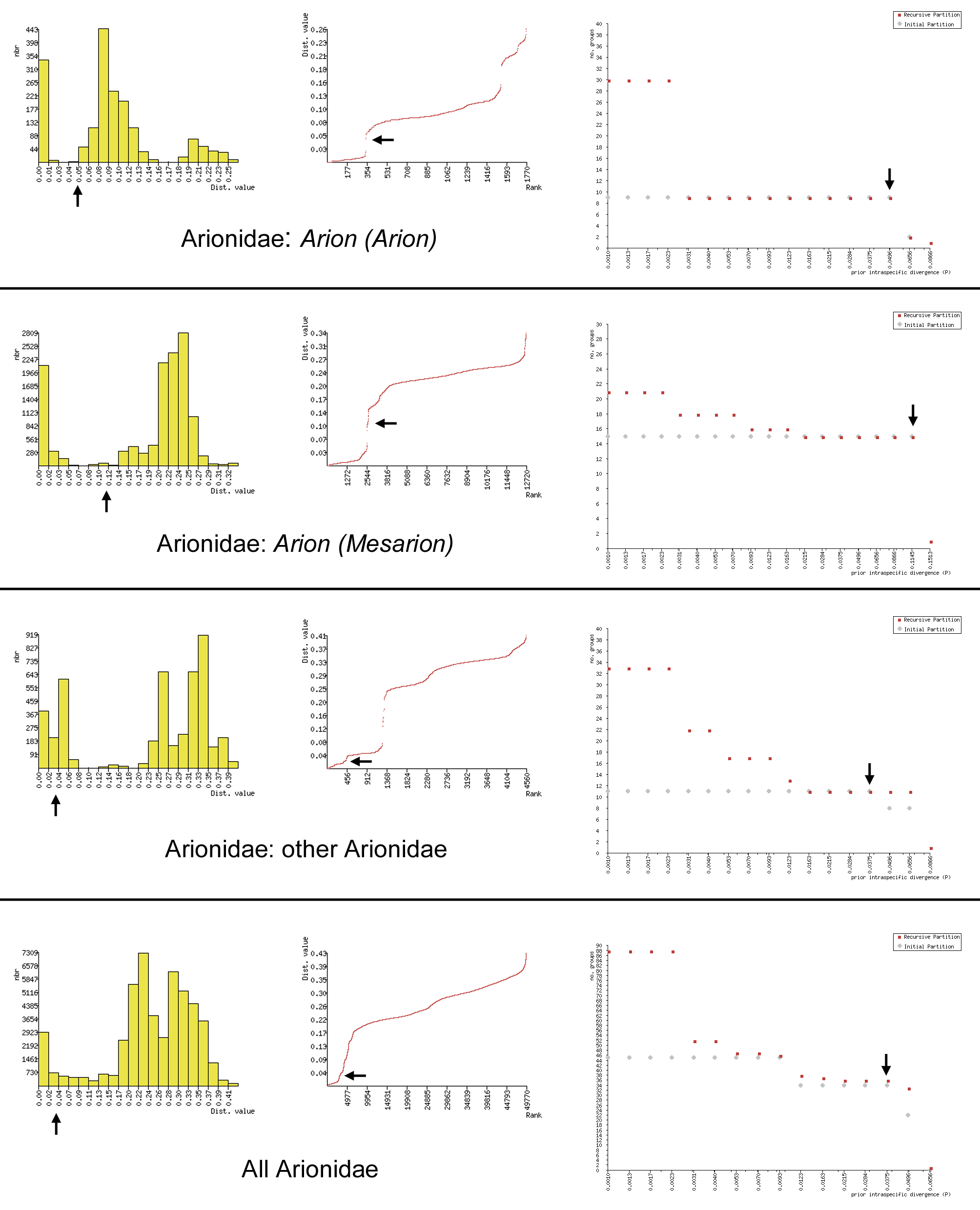

Supplement: Figure S1 — ABGD analysis for Arionidae. Arrow indicates selected PSH and its corresponding position on the distribution of pairwise K2P distances. (TIF) [file pone.0091907.s001.tif]

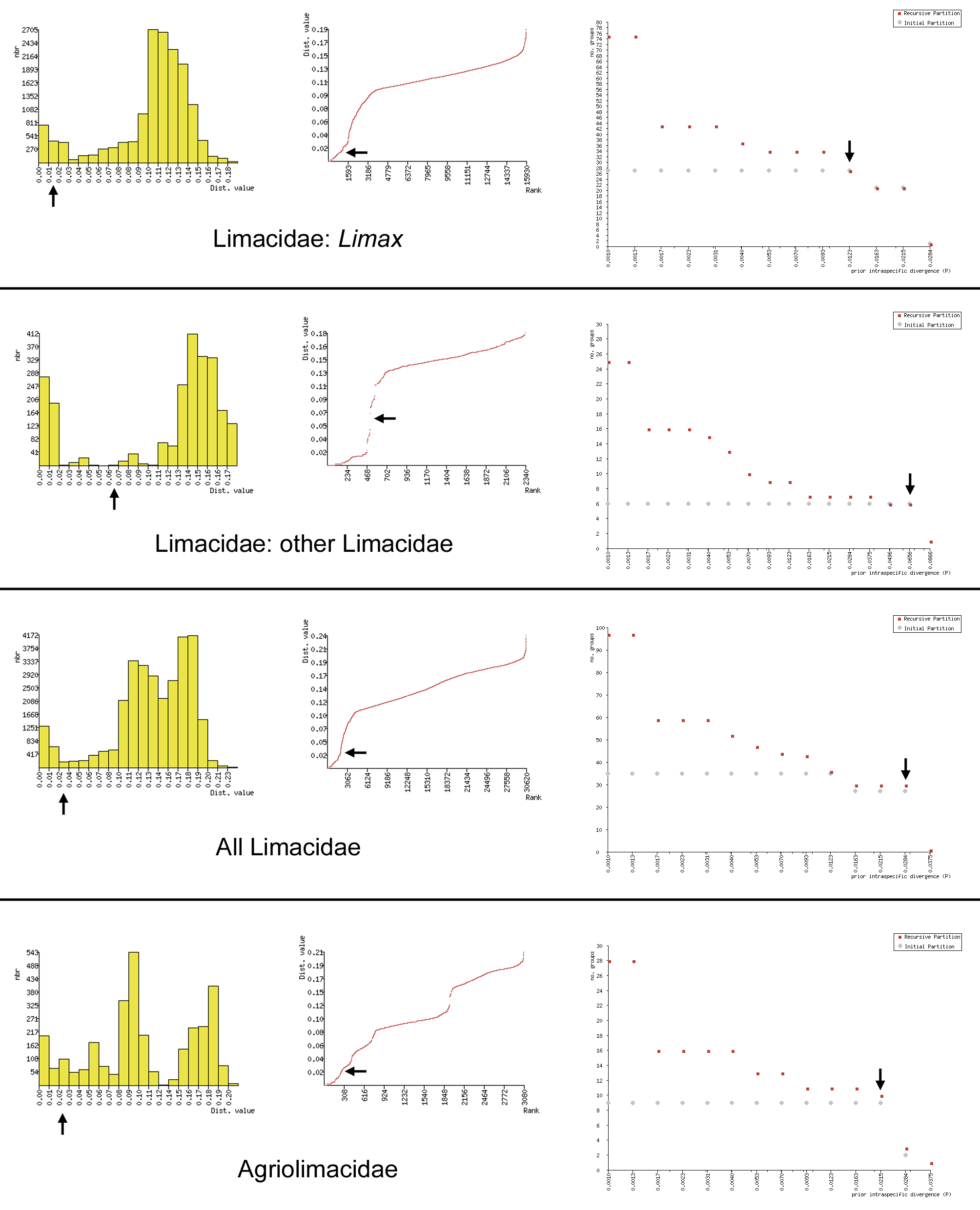

Supplement: Figure S2 — ABGD analysis for Limacidae and Agriolimacidae. Arrow indicates selected PSH and its corresponding position on the distribution of pairwise K2P distances. (TIF) [file pone.0091907.s002.tif]

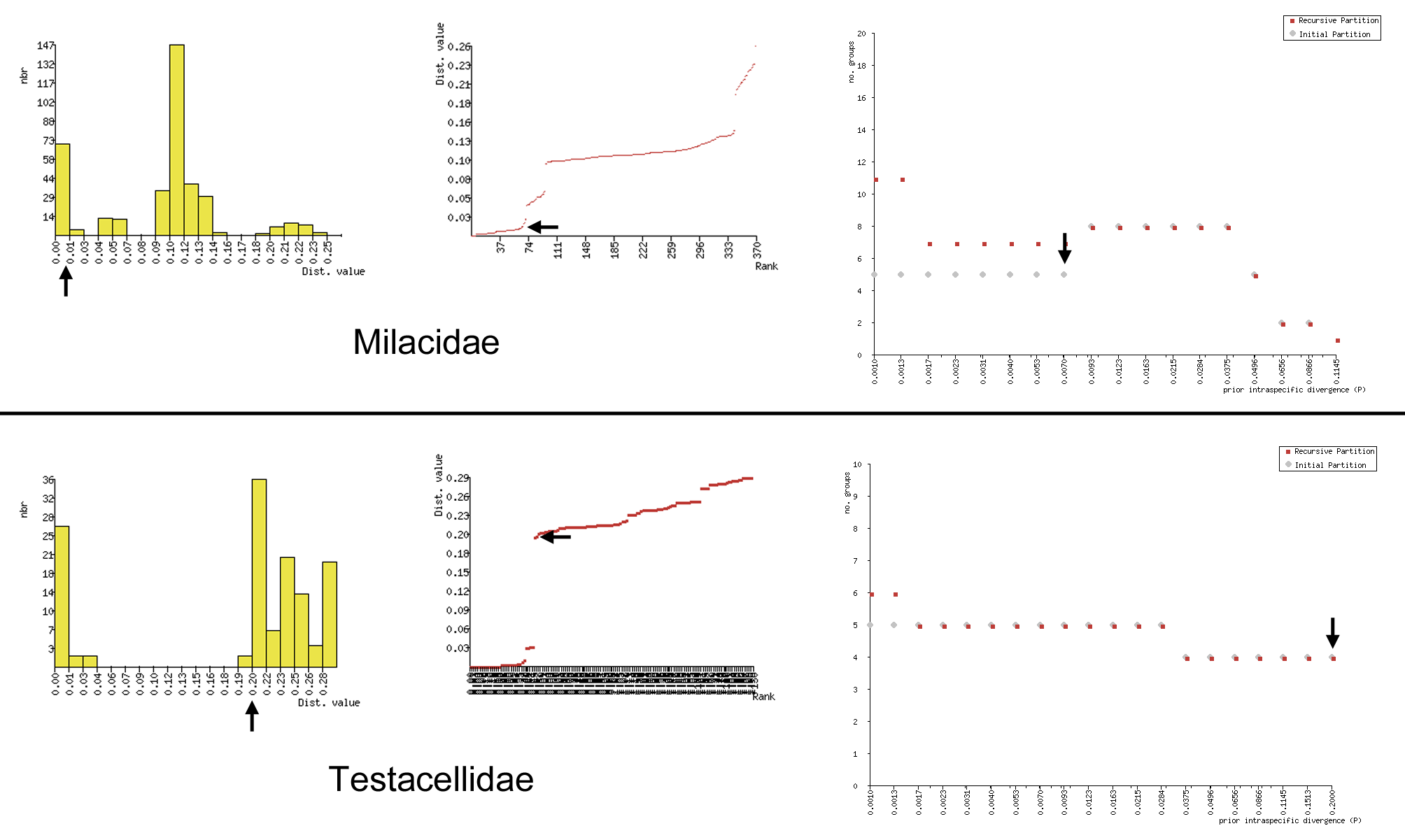

Supplement: Figure S3 — ABGD analysis for Milacidae and Testacellidae. Arrow indicates selected PSH and its corresponding position on the distribution of pairwise K2P distances. (TIF) [file pone.0091907.s003.tif]
