# Supplementary material for: The Slugs of Britain and Ireland: Undetected and Undescribed Species Increase a Well-Studied, Economically Important Fauna by More Than 20%
Source: PLoS One. 2014 Apr 16;9(4):e91907. doi: 10.1371/journal.pone.0091907 (PMC3989179; doi:10.1371/journal.pone.0091907)
Supplement: References S1 — Additional references cited in Table S2. (DOC) [file pone.0091907.s006.doc]

**References S1. Additional references cited in Table S2.**

1. Dvořák L, Backeljau T, Reischütz PL, Horsák M, Breugelmans K, et al. (2006) *Arion alpinus* Pollonera, 1887 in the Czech Republic (Gastropoda: Arionidae). Malacol Bohemoslov 5: 51-55.
2. Pinceel J, Jordaens K, Pfenninger P, Backeljau T (2005) Rangewide phylogeography of a terrestrial slug in Europe: evidence for Alpine refugia and rapid colonization after the Pleistocene glaciations. Mol Ecol 14: 1133-1150.
3. Hyman IT, Ho SY, Jermiin LS (2007) Molecular phylogeny of Australian Helicarionidae, Euconulidae and related groups (Gastropoda: Pulmonata: Stylommatophora) based on mitochondrial DNA. Mol Phylogenet Evol 45: 792-812.
4. Regnier C, Gargominy O, Falkner G, Puillandre N (2011) Foot mucus stored on FTA cards is a reliable and non-invasive source of DNA for genetics studies in molluscs. Conserv Genet Resour 3: 377-382.
5. Roth B, Jadin R, Guralnick R (2013) The taxonomic status of *Deroceras hesperium* Pilsbry, 1944 (Gastropoda: Pulmonata: Agriolimacidae), a species of conservation concern in Oregon, USA. Zootaxa 3691: 453-460.
6. Tsai C-L, Wu S-K (2008) A new *Meghimatium* slug (Pulmonata: Philomycidae) from Taiwan. Zool Stud 47: 759-766.
7. Eskelson MJ, Chapman EG, Archbold DD, Obrycki JJ, Harwood JD (2011) Molecular identification of predation by carabid beetles on exotic and native slugs in a strawberry agroecosystem. Biol Control 56: 245-253.
8. Quinteiro J, Rodríguez-Castro J, Castillejo J, Iglesias-Piñeiro J, Rey-Méndez M (2005) Phylogeny of slug species of the genus *Arion*: evidence of monophyly of Iberian endemics and of the existence of relict species in Pyrenean refuges. J Zoolog Syst Evol Res 43: 139-148.
9. Manganelli G, Bodon M, Giusti F (2010) The status of *Arion alpinus* Pollonera 1887, and re-description of *Arion obesoductus* Reischütz 1973 (Gastropoda, Arionidae). J Conchol 40: 269-276.
